# Emotional disclosure in palliative care: A scoping review of intervention characteristics and implementation factors

**DOI:** 10.1177/02692163211013248

**Published:** 2021-05-29

**Authors:** Daisy McInnerney, Nuriye Kupeli, Paddy Stone, Kanthee Anantapong, Justin Chan, Kate Flemming, Nicholas Troop, Bridget Candy

**Affiliations:** 1 Division of Psychiatry, Marie Curie Palliative Care Research Department, UCL, London, UK; 2 Department of Psychiatry, Faculty of Medicine, Prince of Songkla University, Hat Yai, Thailand; 3 Department of Health Sciences, University of York, York, UK; 4 Department of Psychology, Sports and Geography, University of Hertfordshire, Hertfordshire, UK

**Keywords:** Palliative care, psychotherapy, emotions, mental health, scoping review, Intervention Component Analysis

## Abstract

**Background:**

Emotional disclosure is the therapeutic expression of emotion. It holds potential as a means of providing psychological support. However, evidence of its efficacy in palliative settings is mixed. This may be due to variation in intervention characteristics.

**Aim:**

To derive a greater understanding of the characteristics of potentially effective emotional disclosure-based interventions in palliative care by:

(1) Developing a taxonomy of emotional disclosure-based interventions tested in people with advanced disease and

(2) Mapping and linking objectives, outcomes, underlying mechanisms, and implementation factors.

**Design:**

A scoping review drawing on Intervention Component Analysis to combine evidence from studies’ methods, results, and discussion sections.

**Data sources:**

Six databases were searched to May 2020 including CINAHL, PsycINFO, and MEDLINE. Studies of emotional disclosure in adults with advanced disease were included. Study quality was appraised using an established tool.

**Results:**

Seven thousand seven hundred ninety-two unique records were screened, of which 25 primary studies were included. Intervention characteristics were grouped into classes within three domains: topic of disclosure, format, and dose. Evidence was not available to determine which, if any, of the characteristics is most effective. Thematic synthesis of evidence from methods and discussion sections identified factors to consider in tailoring an emotional disclosure-based intervention to this setting, including: population characteristics (e.g. time since diagnosis), providing a safe environment, and flexibility in format.

**Conclusions:**

This review approach facilitated a clearer understanding of factors that may be key in developing emotional disclosure-based interventions for palliative populations. Intervention Component Analysis has potential for application elsewhere to help develop evidence-based interventions.

**What is already known about the topic?**

- Emotional disclosure-based interventions can improve psychological and physical wellbeing in general populations.
- A range of emotional disclosure-based interventions exist, but evidence of their efficacy in palliative care is mixed; it is not clear in which forms they may be effective or most effective, and on which outcome measures.
- Trials have been limited in the extent to which they have tailored the intervention for people with advanced disease.

**What this paper adds**

- To our knowledge, this is the first scoping review to systematically map the characteristics of emotional disclosure-based interventions that have been tested in people with advanced disease.
- By grouping intervention characteristics into classes within operative domains and mapping these to outcomes, we provide a picture of which intervention forms may be most promising to pursue in future research.
- Disease stage, environment, flexibility in delivery and topic, clarity of instructions, and staff training are identified as important factors to consider when tailoring emotional disclosure-based interventions for people with advanced disease.

**Implications for practice, theory or policy**

- The review provides an exemplar approach to scoping literature to inform complex intervention development and evaluation in cases where pre-existing findings are mixed.
- The review highlights the need for researchers to report key facilitators and barriers they find in intervention implementation and efficacy when presenting results.
- Researchers should consider the recommendations made in this review to inform development and evaluation of emotional disclosure-based interventions tailored for people with advanced disease.

## Introduction

Psychological distress can be considerable for people living with advanced disease. For up to 50% of people receiving palliative care, this distress can develop into clinical anxiety or depression.^1–4^ In recognition of this, national and international clinical guidelines recommend that psychological support should form a crucial element of the holistic palliative care approach.^5–9^ However, research indicates current psychological service provision in palliative care is likely to be inadequate in the UK and globally.^10–12^ This can be partially attributed to limitations in funding for the end-of-life care sector.^12–14^ It is therefore important that palliative care services can access and implement cost-effective ways of providing psychological support for people in their care.

Certain forms of emotional disclosure-based interventions offer a potentially promising solution. For the purposes of this review, emotional disclosure is defined as techniques designed to encourage or facilitate the disclosure, expression or discussion of emotions or feelings. These therapies are based on the notion that expressing emotions can improve wellbeing.^15^ The therapeutic potential of emotional disclosure has been recognized cross-culturally for centuries in the form of religious confessions and Freudian psychotherapeutic approaches.^15^ For example, drawing on this long history, a simple expressive writing intervention was proposed in 1986.^16^ In its most basic format, it involves writing down the facts and emotions about a trauma for 15–20 min per day over 3–4 consecutive days without the need for professional facilitation.^17^ Hundreds of studies have since investigated expressive writing and emotional disclosure-based variations, with meta-analyses reporting small but positive effects on both physical and psychological health in various populations.^18–21^

Trials of emotional disclosure-based interventions in palliative populations, however, have had mixed-results.^22–24^ A recent meta-analysis of randomized controlled trials (RCTs) of expressive writing in people with advanced disease found, overall, it had no significant effect on the physical or psychological health measures investigated.^24^ However, this evidence is weak; it is from four RCTs of limited quality, with only one of these using an intervention that had been specifically tailored to the unique needs of its population.^25^ Whilst this study did individually report a positive effect of the intervention, it was a pilot with 13 participants, and thus was not designed to detect significance.^25^ The importance of tailoring interventions to the target population is likely to be crucial, given the unique existential distress and physical challenges experienced by people at this stage of their illness. As a result, there is still a need for further, robustly designed trials of tailored emotional disclosure-based interventions.

In their guidelines for complex intervention development, the Medical Research Council outline the importance of having a clear theoretical rationale for an intervention and its component parts.^26–28^ A number of processes have been proposed to explain the potential effects of emotional disclosure, including emotion regulation and the psychosomatic theory of inhibition.^29^ However, it is not clear to what extent existing interventions tested in palliative care draw on these processes to inform their design.^29–31^ Forming clearer links between underlying processes and intervention design may also help to inform outcome measure selection. Outside of advanced disease populations, reviews have found significant as well as null effects of emotional disclosure-based interventions on a range of psychological and physical symptoms.^18,31,32^ As such, it is not clear which outcome measures may be most appropriate for evaluating effectiveness.

Moreover, the content and structure of emotional disclosure-based interventions can vary widely, further complicating the evaluation process. For instance, session length, frequency of delivery, and the topic of the disclosure can vary. Emotional disclosure-based interventions also go beyond expressive writing and can include, for example, spoken disclosure,^33^ poetry,^34^ and narrative therapy.^35^ There is often overlap between types of intervention (for example, written and spoken forms) and the language used to describe them. It is therefore challenging to understand which, if any, intervention components may potentially be most effective. To our knowledge, no review to date has explored the range of emotional disclosure-based interventions tested in palliative populations.

In summary, emotional disclosure-based interventions still appear to hold therapeutic potential for people with advanced disease. A lack of clarity on which emotional disclosure-based intervention characteristics may be optimal, their mechanisms of action and appropriate outcome measures, may limit our current understanding of how such interventions may be beneficial for palliative populations.^26,36^ This scoping review therefore aims to derive a greater understanding of the range of emotional disclosure-based interventions evaluated in palliative populations, looking beyond expressive writing, and to understand what a potentially effective one may look like.

The objectives of the review are to:

1. Develop a taxonomy of emotional disclosure-based interventions used for people with advanced disease. The taxonomy will identify, categorize, and define classes (i.e. types) of intervention that fall under the umbrella term “emotional disclosure.”
2. Map and identify any potential links between intervention characteristics, objectives, outcome measures, underlying mechanisms, facilitators and barriers, and efficacy of emotional disclosure-based interventions for people with advanced disease.

## Methods

A scoping review is a suitable method for mapping out complex literature bases in a systematic manner.^37^ This review was conducted in six key stages, guided by standard scoping review frameworks.^38–40^ The protocol guiding this scoping review is reported elsewhere.^41^ In line with the iterative nature of scoping reviews, the protocol has been updated throughout the process, as documented in Supplemental File 1.

### Stage 1. Defining the research question

The following research questions were defined:

1. Which psychotherapeutic interventions for patients with advanced disease are categorized as, or explicitly grounded in, emotional disclosure?
2. What are the primary objectives and characteristics of emotional disclosure-based interventions evaluated in this population?
3. What outcome measures are used to assess the efficacy of emotional disclosure-based interventions in this setting, and which of these captured significant effects?
4. What theoretical frameworks are used to explain the mechanisms underlying emotional disclosure-based interventions in this setting?
5. What are the facilitators and barriers to feasibility and efficacy of emotional disclosure-based interventions in this setting?

### Stage 2. Identifying relevant studies

#### Eligibility criteria

All primary studies (irrespective of design) of emotional disclosure-based psychotherapeutic interventions were included, provided they:

a. Described the method of at least one task or exercise as part of the intervention that is designed to encourage or facilitate the disclosure, expression or discussion of emotions or feelings AND
b. Described emotional disclosure or expression of emotions as a key goal, rationale or functional mechanism of the intervention

Only studies testing interventions with adults (aged 18 and above) with a diagnosis of an advanced disease, such as metastatic cancer (or characterized as Stage III or IV), and/or being explicitly treated with a palliative intent were included. Advanced disease is a broad and commonly used term selected to capture the broad range of diagnoses that could fall under the remit of palliative care. Samples which included >50% patients with advanced disease were also included.

### Exclusion criteria

Publications not in the English language, review articles, discussion pieces, book chapters, and dissertations/theses were excluded. Music, art, life review, dignity, and group therapies were excluded as distinct therapy types that have been reviewed elsewhere.^42–48^

#### Databases

Six databases were searched from inception to May 2020: CINAHL, Cochrane Central Register of Controlled Trials (CENTRAL), PsycINFO, Scopus, Web of Science, and MEDLINE. The European Union Clinical Trials Register, clinicaltrials.gov, the European Association for Palliative Care conference abstracts for the last 7 years (2012–2019) and reference lists of relevant studies, review articles, book chapters, and theses were also checked.

#### Search strategy

A combination of Medical Subject Headings (MeSH) and free-text search terms for emotional disclosure, advanced disease, and palliative care were used. The terms for emotional disclosure were based on earlier, related reviews, but adapted to capture a range of disclosure formats.^22,24^ The terms for advanced disease and palliative care were based on a previous review,^24^ recommended by the Cochrane Palliative Care research group. An example of the search strategy string used for the Ovid PsycINFO database is shown in Table 1. The string was optimized for each database (see Supplemental File 2).

**Table 1.** Search strategy string for PsycINFO database.

exp Emotions/ OR emotion* OR feeling* AND Palliative Care/ OR (palliat* or terminal* or endstage or hospice* or metasta* or (end adj3 life) or (care adj3 dying) or ((advanced or late or last or end or final) adj3 (stage* or phase*))).tw. AND (disclos* or express* or communicat* or talk* or speak* or spoke* or writ* or draw* or sing*).mp.

Filters applied: humans and adulthood (18+).

### Stage 3. Study selection

Two reviewers independently screened titles and abstracts for inclusion to the full article review stage. Full article review was also conducted independently by two researchers. Unclear decisions were discussed between members of the review team.

### Stage 4. Charting the data

A data extraction form was developed based on the variables most relevant to the research questions (see Supplemental File 3). Extraction was completed by one author and a sample of five studies checked by a second author.

### Stage 5. Collating, summarizing, and reporting the results

Synthesis was based on Intervention Component Analysis, which is a pragmatic approach to identifying which characteristics of an intervention, from a group of similar interventions, are potentially important in terms of outcomes.^49^ Intervention Component Analysis uses qualitative thematic techniques to analyze intervention descriptions to identify and group core characteristics of an intervention. Parallel to this, experience-based evidence from study methods and discussion sections is thematically analyzed; this evidence captures authors’ descriptions of their experience developing and implementing the intervention. Whilst Intervention Component Analysis is designed to review interventions reported in trials that aim to influence the same outcome, this scoping review includes a range of study designs using a number of outcome measures. The principles of Intervention Component Analysis were therefore used but the approach was modified to suit the available evidence and meet the review objectives.

After extracting intervention descriptions, through iterative comparison and discussion, three operative domains were identified (i.e. overarching categories within which interventions varied). These were used as a framework for further exploration. Firstly, to form a multi-level taxonomy; using thematic analysis intervention descriptions were coded and similar characteristics grouped into classes (i.e. types) within each of the identified domains (Figure 2). Intervention objectives were then coded and grouped, and these were mapped to the outcome measures being used to assess them (Table 3). Thirdly, intervention classes in the multi-level taxonomy were mapped to the reported efficacy of interventions within them (Table 4). The underlying mechanisms that studies proposed were then grouped into theoretical classes (Table 5). Finally, in parallel to these processes, the facilitators and barriers extracted from discussion sections and methodological descriptions were analyzed using thematic analysis (Figure 3). One author (DM) led the analysis, with themes and conclusions discussed with the research team and updated throughout.

#### Quality appraisal

Study quality was graded by one author using the Hawker tool^50^ and a subset of five was checked by another. Differences were resolved through discussion, and scoring amended as appropriate. In line with the grading used in prior reviews, scores ⩽18 are rated “poor,” scores from 19 to 27 “fair,” and ⩾28 “good.”^51^ Quality appraisal is not a required component of scoping review methodology.^40^ However, as one objective of this review was to map intervention characteristics to their reported efficacy, we recognized a value in assessing the quality of included studies to gauge the reliability of any links drawn from them.

### Stage 6. Consultation

The scoping review was conducted collaboratively at all stages with the core research team, involving a palliative care consultant, a psychiatrist, health psychologist and researchers with expertise in emotional disclosure, palliative care research, and systematic reviewing. Clinical psychologists and a Patient and Public Involvement (PPI) representative were also consulted at key points.

## Results

### Characteristics of included studies

**Figure 1.**
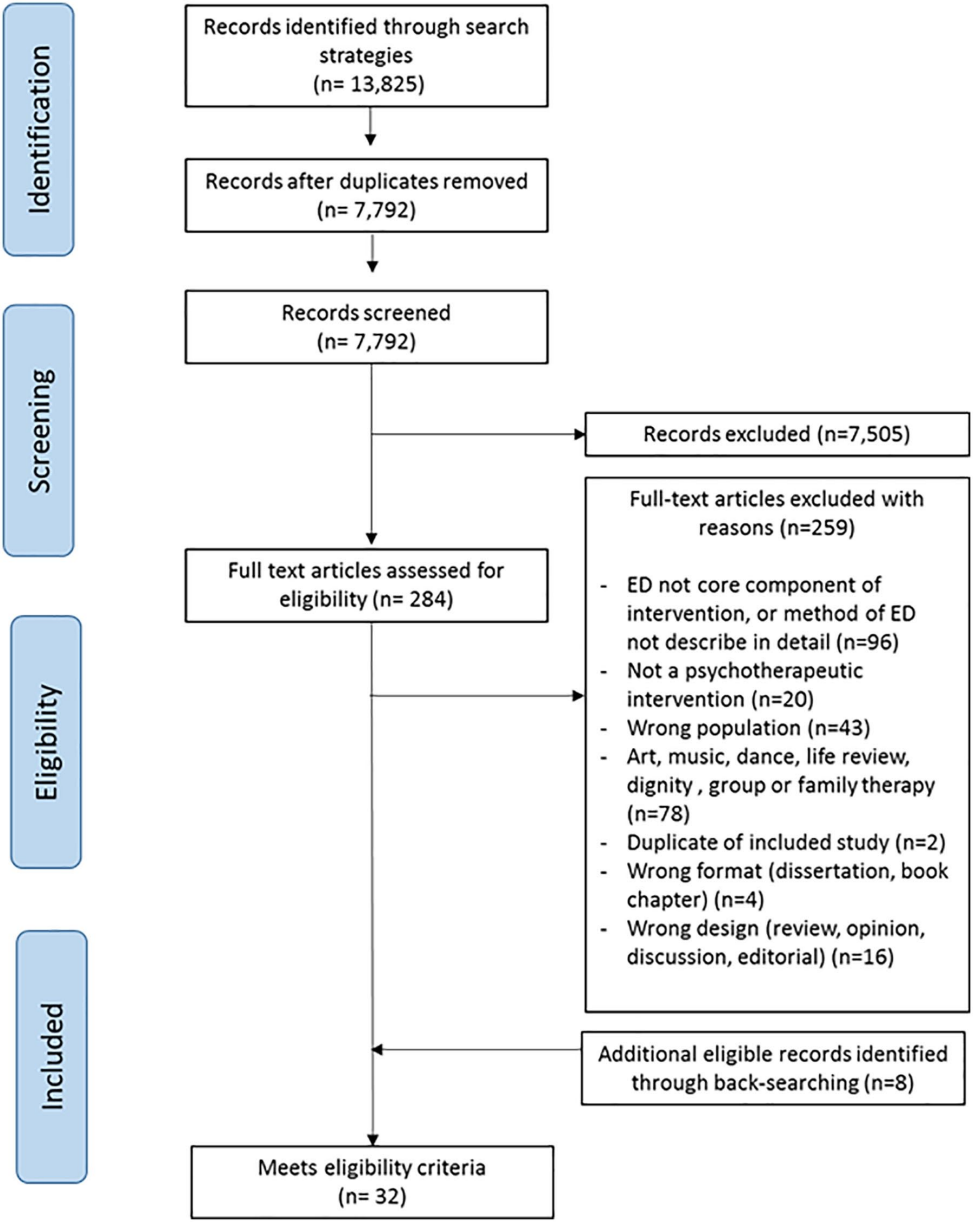
PRISMA diagram of study selection.

The literature search identified 7792 unique citations. Of these, 25 primary studies reported in 32 papers met the inclusion criteria (17 RCTs, 3 other studies reporting preliminary, secondary, or qualitative analyses of data from RCTs, and 5 other studies of different designs). Figure 1 presents a PRISMA flow diagram of study selection. Of the five studies using different designs,^52–56^ three used qualitative methods^52–54^ of which two were case studies;^52,53^ and two used mixed methods.^55,56^ Studies were conducted in four countries: USA (*n* = 18), UK (*n* = 5), China (*n* = 1), and Uruguay (*n* = 1). Most studies tested the intervention in people with advanced or incurable cancer (*n* = 19); other populations were people with amyotrophic lateral sclerosis (ALS) (*n* = 2), end stage renal disease (ESRD) (*n* = 1), and mixed terminal diagnoses (*n* = 3).

Population and intervention characteristics are detailed in Table 2. More detailed study summaries are reported in Supplemental File 4.

**Table 2.** Study and intervention characteristics.

Reference	Description of intervention	Process of development
RCTs		
**Arden-Close et al.** ^60^ *102 women at* ***all stages of ovarian cancer (>50% at Stage III or IV)*** *and their partners recruited via ovarian cancer charity* *UK*	**Expressive writing (for couples)**—based on Guided Disclosure Protocol (GDP)—instructions sent by post, and participant telephoned at designated time to instruct them to start writing, and again 15 min later telling them to stop **Where:** At home **Dose:** 15 min per day over 3 days within the same week (preferably consecutively). Patients and partners could write at the same or different times **Topic:** Patient’s diagnosis and treatment. Day 1: chronological description of event; Day 2: thoughts and feelings at the time of the event; Day 3: how they currently think and feel about the event, and reflections on future coping with a similar event. **Who:** Single researcher	The intervention was based on GDP, a protocol developed by Duncan and Gidron^85^ based on the cognitive processing hypothesis of trauma and tested in rheumatoid arthritis^86^ and fibromyalgia.^87^ **Consultation with stakeholders not reported.**
**Averill et al.** ^33^ *33 males and 15 females with* ***amyotrophic lateral sclerosis (ALS) likely to survive for at least 6 months*** *with good psychological health recruited via ALS registries* *US*	**Written or spoken emotional disclosure**—provided with written instructions for how to complete the exercise, suggestions (e.g. find a quiet place where you can write undisturbed) and paper on which to write and asked to either write (*N* = 10) or talk into a tape recorder (*N* = 8) **Where:** At home **Dose:** 20 min per day for 3 days over the period of a week **Topic:** Deepest feelings and thoughts related to their experience with ALS **Who:** Research nurse	The development process was not reported; a range of EW interventions were cited in background (e.g. ^16,61,87–89^). **Consultation with stakeholders not reported.**
**Bruera et al.** ^66^ *15 females and 9 males with* ***advanced gynecological and prostate cancer*** *referred to palliative care or inpatient unit* *US*	**Expressive writing**—typed or handwritten **Where:** Remote (via phone call) but exact location not reported **Dose:** 20-min writing sessions, twice per week, for 2 weeks **Topic:** Their most upsetting experiences, important things about which they had the deepest feelings and thoughts about their cancer, and an event or experience that they had not talked about with others in detail **Who:** Research nurse	The development process was not reported; cited EW interventions in the background.^61^ **Consultation with stakeholders not reported.**
**De Moor et al.** ^61^ *36 males and 6 females with* ***metastatic Stage IV Renal Cell Carcinoma*** *recruited from a Phase II tumor vaccine trial with life expectancy more than 4 months* *US*	**Expressive writing** **Where:** At each of the first four clinic visits while the patients waited to receive their vaccine treatment. **Dose:** 20 min session once a week for 4 weeks (first four clinic visits as part of trial) **Topic:** To write their deepest thoughts and feelings about their cancer. Specific prompts varied slightly from one session to the next but remained essentially the same. **Who:** Not reported	Writing exercises followed the model developed by Pennebaker and Beall.^16^ **Consultation with stakeholders not reported.**
**Imrie and Troop** ^13^ *8 females and 5 males with* ***life-limiting illness or secondary cancer*** *recruited from a Day Hospice* *UK*	**Compassion-focussed expressive writing (CFEW)** compared to **expressive writing about stress** without compassion instruction (control) **Where:** In a quiet room in the Day Hospice, at the same time as 1–6 other participants **Dose:** Two 20-min sessions, 1 week apart **Topic:** • Control condition—something they found stressful in the last week • Experimental condition—Stress + self-compassion—10 min writing on the stressful event, 10 writing with compassion to the self **Who:** Not reported	**Stakeholders involved in development:** Day Hospice management informed the study design including the spacing of the writing sessions, the writing instructions, the support provided (e.g. pastoral support, informing care staff) and the measures used. This is the reason behind control group task (stress-only): the team felt writing about a neutral topic would be inappropriate.
**Lloyd Williams et al.** ^64^ *68 females and 32 males with* ***advanced metastatic cancer (range of primary sites)*** *at the end stage of their diseases recruited from hospice day units.* *UK*	**Focussed narrative interview.** A random selection were audio-recorded. **Where:** Not reported **Dose:** One off interview delivered at randomization or a few days later if requested. Length of interview not reported. **Topic:** Reflection on sense of meaning, wellbeing and suffering, what they believe to be the main cause of their suffering and any resources they use or professional care provided to maintain their wellbeing. Emphasis on allowing patients to “tell their story.” **Who:** Researcher delivers; training not reported but discussion notes intervention could be delivered by healthcare professionals with “training and supervision”	Narrative therapy, dignity therapy, and supportive-expressive group therapy cited as background.^90–93^ **Consultation with stakeholders not reported.**
**Lloyd Williams et al**.^35^ *39 females and 18 males with* ***advanced cancer receiving palliative care*** *from a hospice day care service with a prognosis between 6 weeks and 12 months, with clinical depression* **UK**	**Focussed narrative semi-structured interview.** A random selection were audio-recorded. **Where:** In hospice or patient’s home **Dose:** One off 25–60 min interview delivered within one week of randomization **Topic:** Sense of meaning regarding distress/depression and physical, psychological and spiritual well-being; what they felt had been the main factor contributing to depression/distress, the resources they had employed, and any medical/professional care received. Emphasis on reflection on inner resources and coping methods. **Who:** Trained researchers with a health background and experience in research with patients with advanced illness	Developed from literature reviews, expert clinician consensus, and pilot work. Drew on Medical Research Council (MRC) framework for the development and evaluation of complex interventions.^26^
**Low et al.** ^58^ *62 women with* ***stage IV metastatic breast cancer*** *receiving any form of treatment, recruited from larger study from oncology clinics, community practices, and online mBC website* *US*	**Expressive writing**—After receiving the written materials, participants call the research office to schedule writing sessions. A trained research assistant telephones the woman at the start of each writing session to read the instructions to the participant, then calls again 20 min later to ask the participant to stop writing. **Where:** At home **Dose:** Four 20-min sessions within a 3-week interval at participant’s convenience **Topic:** Writing about cancer-related emotions **Who:** Trained research assistant	Instructions were adapted from Pennebaker and Beall^16^ and Stanton et al.^94^ The research assistant telephone procedure was based on a protocol followed in previous expressive writing research with cancer patients and loved ones.^95,96^ **Consultation with stakeholders not reported.**
**Manne et al.** ^59^ *253 women with* ***gynecological cancer (>50% advanced)*** *recruited from cancer centers and hospitals* *US*	**Spoken disclosure in Supportive Counselling**—therapist using active but non-directive and non-interpretive techniques to facilitate emotional expression **Where**—At the oncology offices of the study site **Dose**—6 h-long sessions and a phone booster session 1 week after final session **Topic**—Reactions to their cancer **Who**—Trained social workers or psychologists with 5–15 years therapy experience	Components of the Supportive Counselling intervention included those commonly used in Supportive Counselling and Emotion-Focused Therapy techniques. **Consultation with stakeholders not reported.**
**Manne et al.**^75^; **Manne et al.**^76^ ; **Virtue et al.**^77^ ; **Virtue et al.**^80^ *252 women with* ***gynecological cancer (>50% advanced)*** *recruited from cancer centers and hospitals* *US*	**Spoken disclosure in supportive counselling**—as per Manne et al.^59^ but “bolstered by training therapists to facilitate expression of emotional reactions and understanding them” and an additional session **Where**—At the oncology offices of the study site **Dose**—Seven hour-long sessions and a phone booster session 2–3 weeks after final session **Topic**—Experiences with and reactions to their cancer **Who**—Trained social workers, master-level or doctoral-level psychologists, or psychiatrists who were practicing in the community or employees of each cancer centre with between one and 34 years of therapy experience	Components drawn from supportive counselling and emotion-focused therapy, intervention based on SC in Manne et al.^59^ but with adaptations to facilitate emotional expression. **Consultation with stakeholders not reported.**
**Milbury et al.** ^68,74^ *38 females and 37 males with* ***metastatic lung cancer*** *(and their partners) recruited from a cancer centre* *US*	**Online couple-based meditation with spoken emotional disclosure** **Where**—Session 1 is completed face-to-face or online via videoconferences depending on the participants’ availability. Sessions 2–4 are delivered via videoconferencing **Dose**—4 h long sessions over 4 weeks. Additional home materials (CDs, printed materials, exercises) and guidance to disclosure reflections to partner. At least one booster telephone call per week over the 4-week intervention period. The phone call is intended as a homework reminder and addresses any questions regarding the homework. **Topic**—Session 1: Mindful meditation focus (not emotional sharing focus); Session 2: Compassion and positive emotions and emotional disclosure task; Session 3: Gratitude and emotional disclosure; Session 4: Purpose and value-based living **Who**—A trained master’s level mind-body intervention specialist who is experienced in working with cancer patients and their families	Based on mindfulness-based intervention literature for cancer and previous work in patients with stage I–III lung cancer and their partners^97^ and integrating partner-assisted emotional disclosure, citing Porter et al.^62^ **Consultation with stakeholders not reported.** **Authors have conducted pilot work with metastatic lung cancer patients to inform and refine content.**^56^
**Milbury et al.** ^67^ *16 females and 18 males with* ***primary or metastatic brain tumors (>50% advanced)*** *recruited from clinics* *US*	**Online couple-based meditation with spoken emotional disclosure**—**sessions with therapist over FaceTime** **Where**—remote (online), exact location not reported **Dose**—Four weekly (60 min each) sessions. One third of each session dedicated to disclosure/reflection. **Topic**—Session 1: Mindful awareness of experiences; Session 2: Interconnectedness and feelings of compassion to themselves and their partner; Session 3: Things, events and people for which they are grateful; Session4: Value-based living (“What do you want your life to be about?”) **Who**—masters level licensed psychological counsellor intern	Intervention was developed “building on existing evidence”; the emotional disclosure elements based on Porter et al.^62^ partner-assisted emotional disclosure. **Consultation with stakeholders not reported.**
**Mosher et al.** ^57^ *87 women with* ***metastatic (Stage IV) breast cancer*** *attending comprehensive cancer centre with clinically elevated distress* *US*	**Expressive writing**—participants receive written instructions by post and are telephoned by research fellow prior to each session, then phoned back immediately after session. Overview of exercise provided before Session 1. Participants return essays to research team by post. **Where:** At home **Dose:** 20 min of writing, four sessions, over 4–7 weeks **Topic:** Deepest thoughts and feelings regarding the cancer **Who:** Research fellow	Participants followed the protocol used by Zakowski et al.^98^ for written emotional disclosure in cancer patients. **Consultation with stakeholders not reported.**
**Porter et al.** ^62^ *92 males and 38 females with* ***gastrointestinal cancer (>50% advanced)*** *(and their partners) recruited from hospital oncology clinics* *US*	**Partner-assisted emotional disclosure—**participants attend sessions with a trained therapist who guides the patient to describe the events and their feelings about a cancer-related experience that caused strong emotions; the partner is trained to listen supportively and receptively. **Where:** At the medical centre (although encouraged to continue the discussions at home) **Dose:** Four weekly sessions (session 1, 75 min; sessions 2–4, 45 min) spread over up to 8 weeks **Topic:** The events and feelings about cancer-related experiences that caused strong emotions **Who:** Trained master’s level therapist (social worker or psychologist)	A novel intervention building on private emotional disclosure and the cognitive-behavioral marital literature. **Consultation with stakeholders not reported.**
**Steinhauser et al.** ^72,73^ *38 female and 44 male hospice patients with* ***varying diagnoses and a prognosis of less than 6 months*** *to live, recruited from inpatient and outpatient hospital, palliative care, and hospice settings* *18 took part in qualitative interviews (2009).* *US*	**Spoken disclosure (Outlook intervention)** in semi-structured, audio-recorded interview. At the end of each session, participants were given a handout, printed on cardstock, which explored the session content to seed further reflection **Where:** In participants’ homes **Dose:** Three 45 min–1 h interviews, 1 week apart **Topic:** Issues related to life completion and preparation: Session 1: life review, accomplishments, proudest moments, and cherished times Session 2: issues of forgiveness, things they would have done differently, things left unsaid or undone. Session 3: lessons learned, heritage, and legacy **Who:** Research assistant trained not to give implicit or explicit messages or agenda of specific content/emotional disclosure	Linking life review, emotional self-disclosure, and social gerontology literatures to inform development. **A team of clinician and non-clinician researchers developed and refined Outlook’s content and structure.**
**Steinhauser et al.** ^63^ *212 male and 9 female* ***hospice ineligible advanced disease patients*** *(to understand benefits in early palliative care context) recruited from outpatient clinics* *US*	**Spoken disclosure (Outlook intervention)**—as in Steinhauser 2008; 2009 **Where:** Not reported **Dose:** 3 interviews over the space of 1 month (typically 1 week apart). **Topic:** As in Steinhauser 2008; 2009 **Who:** Clinical social worker following manualized script and receiving ongoing supervision	*As in Steinhauser 2008;2009*
**Zhu et al.** ^65^ *6 males and 10 females with* ***incurable cancer*** *recruited from cancer clinics* *US*	**Written disclosure in Creative Writing Workshops (“Write from the heart”)** **Where:** Not reported **Dose:** 2-h long weekly CWW × 4 weeks **Topic:** Express their feelings about random things in life and was not restricted to cancer-related topics **Who:** Professional writer	Not reported; cite creative writing studies and workshops in the background. **Consultation with stakeholders not reported.**
Secondary analyses of RCTs		
**Laccetti et al.** ^79^ *Descriptive, correlational secondary analysis of RCT* *68 women with* ***metastatic breast cancer and life expectancy >6 months*** *recruited from medical centers, community centers, and private clinic* *US*	**Written disclosure** **Where:** Place and time of participant’s choosing **Dose:** Writing for 20–30 min a day for four consecutive days **Topic:** Writing about experiences with metastatic breast cancer, thoughts, and feelings related to not fully recovering from cancer and facing death, and any other traumatic and upsetting experiences in life that may or may not relate to breast cancer. **Who:** Not reported; alludes to being an intervention that can be prescribed and guided by nurses	Based on Pennebaker’s expressive writing/facilitated disclosure and cite studies that have used EW in people with cancer (non-advanced).^16^ **Consultation with stakeholders not reported.**
**Leal et al.** ^78^ *Qualitative evaluation of EW texts from RCT* *16 females and 21 males with* ***renal cell carcinoma recruited*** *from RCT of EW in people with renal cell carcinoma of all stages* *US*	**Written disclosure** **Where:** Participants’ home **Dose:** Four 20 min writing sessions over a 10 days period; between 1 and 4 days between sessions **Topic:** Writing about illness and other fears in response to four prompts: 1: First told you had cancer or about making decisions about your treatment 2: Adjusting to your cancer, how it has changed your life, or how it has affected your family 3: Fears, worries and concerns you may be experiencing 4: Thoughts and feelings about the future, or your fears and worries about the treatment not working **Who:** Research assistant (training not reported)	Pennebaker and Beall’s intervention informed the general writing procedures.^16^ **Pilot work with cancer patients informed modifications of the intervention.**^61,98,99^
**Rose et al.**^69,71^; **Radziewicz**^70^ *Evaluation of RCT* *110 males and 51 females with* ***advanced cancer*** *(median life expectancy of one year or less) recruited from two ambulatory cancer clinics* *US*	**Spoken disclosure** via telephone-based Coping and Communication Support (CCS) intervention **Where:** Flexible as telephone-based **Dose:** Flexible and tailored to patient preference (all receive an initial phone call within 2 weeks of initial consultation; monthly calls recommended for those with high levels of distress; CCS Practitioners on call 24/7 to take calls) **Topic:** Patient concerns (psychological, existential, practical, symptoms, caregiver burden) and communication issues (family and friends, healthcare providers) **Who:** Advanced practice nurses with mental health training (CCS Practitioners)	Based on review of psycho-oncology interventions, including SUPPORT intervention (nurse discussions with patients and families about care decisions) and the informing theoretical frameworks.^100^ **Stakeholder consultation not reported**
Non-RCTs		
**Garcia Perez and Dapueto** ^52^ *Case study* *Female with* ***advanced ALS*** *Uruguay*	**Spoken disclosure via computer-assisted psychotherapy** **Where:** Initially psychologist’s office, moving to patient’s home after 3 months **Dose:** Around 1 h, once a week, starting 4 months after diagnosis **Topic:** General trauma, client’s choice **Who:** Psychotherapist	This psychotherapeutic approach was based on cognitive-behavioral and expressive supportive models and techniques. The technology is an adaptation of augmentative-alternative communication technologies to enhance patient’s speaking capabilities to facilitate psychotherapy. **Stakeholder consultation not reported**
**Milbury et al.** ^56^ *5 women and 8 men with primary or metastatic non-small cell lung cancer recruited from clinics* *US*	**Couple-based meditation with spoken emotional disclosure** **Where**—Clinical consultation room in cancer centre **Dose**—Four hour long sessions over 2 weeks. Additional home materials (CDs, printed materials, exercises) also provided. **Topic**—Session 1: Mindful meditation focus (not emotional sharing focus); Session 2: Connection and loving-kindness for positive emotions; Session 3: Gratitude; Session 4: Purpose and value-based living **Who**—Master-level mind body specialist	Based on principles of interdependence theory, mindfulness-based intervention literature, and related interventions developed for people with stage I–III lung cancer.^97^ **Intervention content evaluation part of study used to refine intervention based on participants’ written and oral feedback.**
**Pon et al.** ^53^ *Case studies* *5 hospice patients with* ***terminal stage cancer with <6 months to live*** *recruited from a hospice program* *China*	**Spoken disclosure** in context of playing **“My Wonderful Life” (MWL) board game**—participant moves along game board performing acts or picking an “Honest expression” card **Where:** Not reported **Dose:** Three sessions, each session 60–90 min long **Topic:** Life review and death preparation components: Making plans for leaving family members, saying final farewells, asking for forgiveness, showing appreciation for and leaving messages for others, recollections of personal contributions, strengths and wisdom, and contextualized perceived failures. **Who:** Therapist/facilitator	Adapted from communication games used in other settings (trauma, pediatric populations).^101,102^ **Stakeholder consultation not reported**
**Taylor et al.** ^54^ *Qualitative evaluation* *24 male and 12 female patients with* ***end-stage renal disease*** *recruited from routine outpatient clinic* *UK*	**Spoken disclosure in response to either:** **a. Patient Issues Sheet (*n*** **=** **21)** for participants to circle 2–3 main issues to discuss during consultation (Intervention 1) or **b. Direct well-being question adapted from PHQ-9 (*n*** **=** **20)** (Intervention 2) **Where:** During consultations **Dose:** Single consultation **Topic:** Intervention 1: Emotional concerns related to illness that they would most like to talk about Intervention 2: Issues experience during the last week **Who:** Renal consultants who have completed training with renal psychologists covering motivational interviewing, open questions, affirmation and reflection, and three stage model of counselling	Development process not reported. **Consultation with stakeholders not reported.**
**Tuck et al.** ^55^ *Mixed methods* *4 male and 3 females with a* ***diagnosis of terminal cancer*** *recruited from palliative care unit* *US*	**Narrative storytelling (spoken, audio-recorded and transcribed)** through the PATS (Presence, Active Listening, Touch, Sacred story) intervention. **Where:** Private room in the palliative care unit or at home **Dose:** One interview spread over several 20–30 min sessions over 8–24 h (flexible depending of patient schedule and health) **Topic:** Experience of finding out they have cancer and there is no treatment or cure. If the following topics are not covered in the resulting story, probes were used exploring spirituality, sacred stories, healing, and change and growth **Who:** Initially by the principal investigator at the palliative care unit and latterly by a doctoral student trained in the protocol	Developed by the first author/principal investigator based on spirituality and healing literature. **Consultation with stakeholders not reported.**

CCS: coping and communication support; CFEW: compassion-focused expressive writing; EMO: emotional writing condition; EW: expressive writing; GDP: guided disclosure protocol; mBC: metastatic breast cancer; MWL: my wonderful life; NW: neutral writing; PATS: presence, active listening, touch, sacred story; PCU: palliative care unit; RCT: randomized controlled trial; RM: relaxation meditation; UC: usual care.

#### Quality appraisal

Of the 32 included papers, 20 were rated as “Good” and 10 as “Fair”; two were not in appropriate formats for quality appraisal (one protocol and one abstract). Supplemental File 5 presents a summary of ratings.

### Multi-level taxonomy of emotional disclosure-based interventions

**Figure 2.**
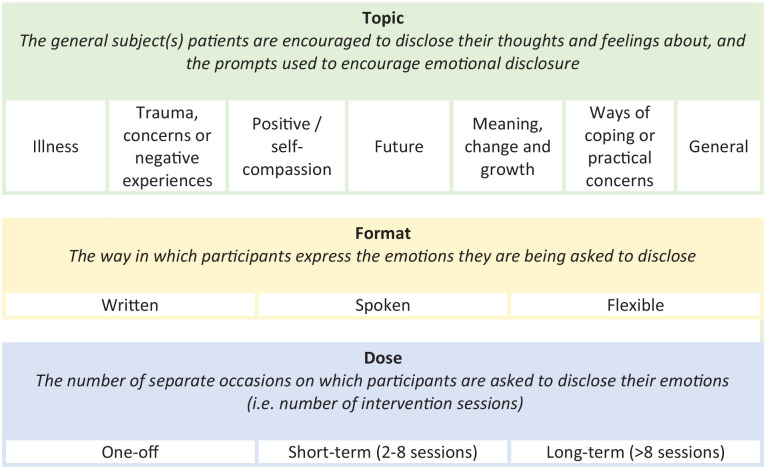
Proposed multi-level taxonomy of ED-based intervention features.

A multi-level taxonomy of emotional disclosure-based interventions is presented in Figure 2. Through iterative discussion and comparison, *topic of disclosure, format of disclosure*, and *dose* were identified as operative domains. Classes are proposed within each domain.

### Primary objectives and outcome measures

Intervention objectives were grouped into the following classes (see Table 3): quality of life, care quality and access, psychological wellbeing, physical wellbeing, existential and spiritual wellbeing, sleep and fatigue, and interpersonal. In cases where studies did not explicitly state primary intervention objectives, the stated aim of the study was used. Classes were then mapped to study primary outcome measures. The most commonly explored class of primary objective (in 14 of 17 RCTs) was psychological wellbeing. Within that class, objectives and outcome measures varied, including a range of anxiety, depression, and overall distress measures. Across the studies, 41 different outcome measures were used to evaluate primary intervention objectives, and follow-up time-points ranged from immediately to 18 months post-intervention.

**Table 3.** Classification of intervention objectives and outcome measures in RCTs.

Class of intervention objective	Primary outcome measures to evaluate objective*	Qualitative assessment methods
Quality of life	Global suffering VAS, QUAL-E, FACT-G, FACT-B, SDS, MDASI-BT/LC	n/a
Care quality and access	Use of mental health services measure	Interview
		Case report
Psychological wellbeing	**General:** DT, ETS, POMS, POMSSF, composite measure, SDHS	Analysis of expression texts
	**Depression:** CES-D, CES-D short version, PHQ-9, BDI	
	**Anxiety:** HADS-A, POMS Anxiety sub-scale, STAI	Interview
	**Trauma and stress:** IES	Case report
	**Stress:** PSS	
	**Self-compassion:** SCS	
	**Other:** FSCRS, SISE, FACT-G (emotional wellbeing subscale); CAR; MAAS	
Physical wellbeing	**Symptom scales:** SSS, MSAS, ADLS, IADLS, ESAS	Analysis of expression texts
Existential and spiritual wellbeing	**Spiritual:** FACT-Sp, DSES, SHI	Analysis of expression texts
	**Preparation and completion:** QUAL-E—preparation and completion sub-scale	Interviews
Sleep and fatigue	**Sleep:** PSQI	n/a
	**Fatigue:** FACT-F	
Interpersonal	**Romantic relationships:** MSIS, QMI, PAIRI	n/a
	**Social support:** brief family social support measure	

BDI: beck depression inventory; CAR: concerns about recurrence subscale; CES-D: center for epidemiological studies depression scale; DSES: daily spiritual experience scale; DT: distress thermometer; ETS: emotion thermometer scale; ESAS: Edmonton symptom assessment scale; FACT: functional assessment of cancer therapy; FACT-B: FACT-breast cancer; FACT-E: FACT-existential; FACT-G: FACT-general; FACT-Sp: FACT-spiritual; FSCRS: forms of self-criticizing and -reassuring scale; HADS: hospital anxiety and depression scale-anxiety; (I)ADLS: (instrumental) activities of daily living scale; IES: impact of events scale; MAAS: mindful-attention awareness scale; MSAS: memorial symptom assessment scale; MSIS: miller social intimacy scale; MDASI-BT: MD Anderson symptom inventory-brain tumor; MDSAI-LC: MD Anderson symptom inventory-lung cancer; PAIRI: personal assessment of intimacy in relationships inventory; PHQ-9: personal health questionnaire-9; POMS: profile of moods scale; POMSSF: POMS short form; PSQI: Pittsburgh sleep quality index; PSS: perceived stress scale; QMI: quality of marriage index; QUAL-E: quality of life at the end of life; SCS: self compassion scale; SDHS: short depression-happiness scale; SDS: symptom distress scale; SHI: self harm inventory; SISE: single item self-esteem scale; SSS: somatic symptom scale; STAI: state trait anxiety inventory; VAS: visual analog scale.

* Some studies’ primary objective were grouped under more than one theme and therefore feature in more than one class.

Significant positive effects were reported in RCTs for 17 different outcome measures (summarized in Table 4); at least one measure within each objective class reported a significant positive effect. However, results using each measure were not consistent across studies. Results are described as “effective” based on statistical significance, although it is recognized that this is limited in that it provides no indication of study quality or effect size. However, what is sought is consistency in findings across studies to guide the direction of future research, rather than making clinical recommendations. All study results are summarized in Supplemental File 4.

**Table 4.** Mapping intervention domains and classes to study outcomes.

Intervention characteristics		Impact of intervention in controlled studies on:*							Summary of qualitative evidence on acceptability and experience
Domain	Class	Quality of life	Psychological wellbeing	Physical wellbeing	Care quality/access	Existential/spiritual wellbeing	Sleep/fatigue	Social	
		Outcome measures for which significant positive effects reported (follow up time-point post-intervention, weeks)							
Topic	Illness	**+**	**+++ OOOOO**	**+**	**+**	**O**	**+ O -** **	**+**	• One qualitative study reported the intervention was well-received and helped patient feel more cared for^54^
		FACT-G (12)	Composite (12) IES (12) BDI (36)	SSS (12)	Uptake MHS (8)		PSQI (up to 10)	MSIS, QMI (0)	
	Trauma	**OO**	**OO**	**O**		**+O**			• One study reported participants found the intervention “overwhelmingly” helpful and could relate as a whole person.^63^ • Two case studies reported positive feedback from participants, including better symptom control, improved communication, reduced distress and promoted dignity and self-esteem^52^ and a sense of release, closure and distraction, as well as facilitating patients entering into therapy.^53^
						QUAL-E (5)			
	Positive	**+OOO**	**++OO**	**O**		**+OOO**	**O**	**+**	• One study reported participants found the intervention “overwhelmingly” helpful and could “relate as a whole person” (i.e. more than just their condition).^63^
		MDASI (2-8)	CES-D, IES (4-12) SCS (2-8)			QUAL-E (5)		PAIRI (2-8)	
	Future	**+**	**O**						• One case study reported positive feedback from participants including a sense of release, closure and distraction, and facilitated patients entering into therapy.^53^
		FACT-G (12)							
	Growth	**+OOOO**	**+++OOO**	**+ OO**		**+ OO**	**OOO**	**+**	• One study reported participants found the intervention “overwhelmingly” helpful and could “relate as a whole person” (i.e. more than just their condition).^63^
		MDASI (2-8)	PHQ-9 (6) CES-D, IES (4-12) SCS (2-8)	ESAS-pain (8)		QUAL-E (5)		PAIRI (2-8)	
	Ways of coping	**O**	**+ O**	**+ O**			**OO**		• One qualitative study reported the interventions were well-received and helped patient feel more cared for^54^
			PHQ-9 (6)	ESAS-pain (8)					
	General	**+O**	**+++**			**O**	**O**	**+**	None reported
		MDASI (2-8)	CES-D, IES (4-12) SCS (2-8) ETS—anxiety (0)					PAIRI (2-8)	
Format	Spoken	**+OOOO**	**++++ OOOOO**	**+OOO**		**+ OO**	**OOO**	**++**	• One study reported participants found intervention “overwhelmingly” helpful^63^ and another reported the interventions were well-received and helped patient feel more cared for ^54^ • Two case studies reported positive feedback from participants, including better symptom control, improved communication, reduced distress and promoted dignity and self-esteem^52^ and a sense of release, closure and distraction, as well as facilitating patients entering into therapy.^53^
		MDASI (2-8)	PHQ-9 (6) BDI (36) CES-D, IES (4-12) SCS (2-8)	ESAS-pain (8)		QUAL-E (5)		PAIRI (2-8) MSIS, QMI (0)	
	Written	**+**	**++ OOO**	**+**	**+**	**O**	**+ O -** **		None reported
		FACT-G (12)	IES (12) ETS—anxiety (0)	SSS (12)	Uptake MHS (12)		PSQI (up to 10)		
	Flexible		**+**						None reported
			Composite measure (12)						

Dose	One-off	**O**	**+ O**	**+ O**			**OO**		• One qualitative study reported the interventions were well-received and helped patient feel more cared for^54^
			PHQ-9 (6)	ESAS-pain (8)					
	Short term	**++ OOO**	**++++++OOOOOOO**	**+O**	**+**	**+ OOO**	**+ OO -** **	**++**	• One study reported participants found intervention “overwhelmingly” helpful and could “relate as a whole person” (i.e. more than just their condition)^63^ • One case study reported positive feedback from participants: a sense of release, closure and distraction, facilitation to enter therapy.^53^
		FACT-G (12) MDASI (2-8)	Composite measure(12) IES (12) BDI (36) CES-D, IES (4-12) SCS (2-8) ETS—anxiety (0)	SSS (12)	Uptake MHS (8)	QUAL-E (5)	PSQI (up to 10)	PAIRI (2-8) MSIS, QMI (0)	
	Long-Term								• One case study reported positive feedback from participant, including better symptom control, improved communication, reduced distress, and promoted dignity and self-esteem^52^
						

Key: +Study reported a significant difference between intervention and control group in favor of intervention on at least one measure in class.

−Study reported a significant difference between intervention and control group in favor of control on at least one measure in class.

O study reported no significant difference between intervention and control group on any measure in class.

The number of +/− in each column indicates the total number of studies that reported a significant difference between the intervention and control group in each class. For measures where a significant difference was identified in favor of the intervention (+), the outcome measures for which those differences were identified are listed. Measures used in the same study are separated by commas. Measures used in different studies are on separate lines.

BDI: beck depression inventory; CES-D: center for epidemiological studies depression scale; ESAS: Edmonton symptom assessment scale; ETS: emotion thermometer scale; FACT-G: functional assessment of cancer therapy- general; IES: impact of events scale; MDASI: MD Anderson symptom inventory; MHS: mental health services; MSIS: miller social intimacy scale; PAIRI: personal assessment of intimacy in relationships inventory; PHQ-9: personal health questionnaire-9; PSQI: Pittsburgh sleep quality index; QMI: quality of marriage index; QUAL-E: quality of life at the end of life; SCS: self-compassion scale; SSS: somatic symptom scale.

* We describe results as effective based on statistical significance reported in the study, although we recognize that this is limited in that it provides no indication of the size or importance of an effect. Detailed results on the nature of the effect reported in each study are reported in Supplemental File 5. Only studies that were designed to evaluate efficacy were included in this part of the table.

** EW participants with a longer duration of time since diagnosis exhibited increases in sleep disturbances.

### Mapping intervention classes to efficacy

Table 4 shows the mapping of classes within each domain in the taxonomy to study outcomes.

#### Topic of disclosure

In the majority of studies, participants were directed to express their feelings about their illness as at least one of the disclosure topics (*n* = 14). Of these, nine were trials, of which six reported significant positive effects on at least one outcome compared to control, including accessing mental health services,^57^ psychological wellbeing,^33,58,59^ quality of life,^60^ sleep,^61^ physical symptoms,^61^ and interpersonal relationships.^62^ One RCT reported a significant negative effect of the intervention which directed people to express emotions about their illness.^58^ This study found that there was a significant interaction between time since diagnosis and group: women in the intervention group with a longer time since diagnosis were more likely to report increased sleep disturbances at 3 months follow-up compared to those in the control group.

Six trials investigated interventions using general trauma or negative experiences as at least one of the disclosure topics. Of these, studies reported a significant improvement in existential and spiritual wellbeing,^63^ pain,^64^ depressive symptoms, and anxiety^65^ compared to control. Two did not find any significant effects on any measure (although they were not powered to do so).^25,66^ Some interventions also asked people to express feelings on growth, ways of coping or positive emotions. Of these, significant positive effects versus control were reported on measures of quality of life,^60,67^ psychological wellbeing,^35,67,68^ physical wellbeing^64^ and existential wellbeing,^63^ as well as interpersonal relationships.^67^ Most interventions asked participants to express feelings about a combination of different topics. In sum, no single topic or combination of topics was consistently related to a positive effect on any particular outcome.

#### Format of disclosure

Most studies (*n* = 15) investigated interventions asking people to express thoughts and feelings through spoken disclosure.^35,52–56,59,62,63,67–77^ Eight studies investigated written disclosure.^57,58,60,61,65,66,78,79^ Two studies explored flexible interventions, which gave participants the option of whether to speak or write,^25,33^ Table 2 gives a description of the nature of these interventions.

RCTs testing spoken interventions reported significant effects on quality of life,^67^ depression,^35^ cancer-related distress,^68^ pain,^64^ self-compassion,^67^ existential/spiritual wellbeing,^63^ and interpersonal relationships.^62,67^ Five RCTs investigating written interventions also reported significant effects compared to control on anxiety,^65^ sleep,^61^ uptake of mental health services,^57^ intrusive thoughts,^58^ somatic symptoms,^58^ and quality of life.^60^ Of the two RCTs that investigated a flexible intervention, one reported a significant improvement in psychological wellbeing 3 months post-intervention.^33^ The other was a feasibility study not designed to evaluate efficacy.^25^ In sum, there were no obvious patterns: all formats resulted in benefits in some outcomes.

#### Dose of disclosure

The majority of studies (*n* = 19) investigated short-term interventions (classified as 2–8 sessions) delivered over a time period of up to 2 months.^25,33,53,56–63,65–68,72,73,75,78,79^ Four studies investigated one-off interventions, two of which were RCTs that reported significant improvements in the emotional disclosure group compared to control (one on pain and one on depression).^35,64^ The other two studies (one case study, and one that did not report on efficacy) investigated longer term interventions delivered on an ongoing weekly or monthly basis and no defined number of sessions.^52,69–71^ Session length as well as the interval between sessions varied considerably (see Table 2). In sum, no links could be made between intervention dose and effectiveness. However, some studies did suggest that for interventions linking emotional processing and awareness to outcomes, more sessions over a longer time period may be needed to produce long-term effects.^59,76,77^

### Overview of underlying mechanisms

The theories and models used to inform intervention development and explain potential effects are summarized in Table 5. Studies drew on a range of communication, social, psychoanalytic, cognitive, developmental and self-compassion theories, but rarely provided a full theoretical justification for each intervention characteristic. One found that low levels of emotional support and more recent diagnoses were associated with better responses to the intervention.^58^ Another found that emotional disclosure increased quality of life only if illness-related couples’ communication also improved.^60^ Studies reported contrasting findings relating to the role of natural expressivity. One study found high levels of baseline emotional expressivity were associated with a larger effect on depressive symptoms.^59^ Others found high levels of holding back,^62^ and ambivalence over emotional expression^33^ were associated with larger effects. In sum, a number of studies investigated moderators of intervention effects to explore underlying mechanisms, with overall mixed findings.

**Table 5.** Theoretical frameworks and underlying mechanisms of emotional disclosure-based interventions.

Theories	Models and/or mechanisms
Communication	Patient–clinician communication models^54,55^
Social and interpersonal	Social constraints inhibiting social-cognitive processing^78^ and communication^68^
	Social integration and interaction models^33,60,57,68^
	Therapeutic value of game play^53^
	Supportive-expressive models^52^
	Interdependence theory^33^
	Intimacy and relationship satisfaction^62,67^
Psychoanalytic	Inhibition and catharsis^53,55,79^
Cognitive	Cognitive-processing mediation model and reappraisal models^25,78^
	Social constraints inhibiting social-cognitive processing^78^
	Emotion regulation^57^
	Emotional processing and awareness^59,76,77^
	Information processing theories^69,71^
	Cognitive-behavioral models^52^
Life-stage and developmental	Continuity in chaotic illness model^78^
	Biographical disruption model/reconstruction of personal narrative^63,72,73,78^
	Health within illness model^78^
Self	Self-compassion^25,67,74^
	Self-regulation of attention^67^
	Self-efficacy and enablement^54,60,69,71^
	Non-directed client-centered approach^69,71,75^
	Ego-functioning, self-esteem, and tolerance of negative affect^59^

### Facilitators and barriers to feasibility and efficacy

**Figure 3.**
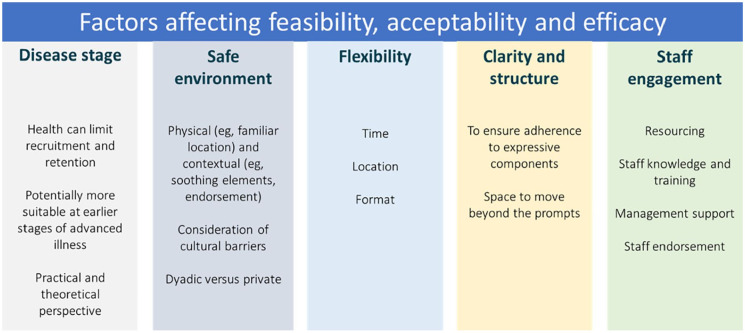
Results of thematic analysis of implementation factors.

This section reports the results of the thematic analysis of experience-based evidence where authors discuss their findings in relation to their intervention design and implementation. We identified five inter-related themes as important factors to consider in development of emotional disclosure-based interventions for palliative populations. These are summarized in Figure 3 and described below.

#### Impact of disease stage and type

Whilst all studies recruited people with advanced disease, the stage ranged from pre-palliative^63^ to people receiving inpatient hospice care with less than 6 months to live.^53,72,73^ Participant health was often noted by authors as a factor limiting recruitment, retention, and adherence.^25,33,35,55,60–62,64,66,72,73^ Some suggested that emotional disclosure-based interventions may be more suitable for people at the earlier stages of advanced illness, as they may be more physically able to complete the intervention.^25,66,72,73^ Some study authors also suggested that emotional disclosure-based interventions may be more suitable for people who have not yet processed the trauma they are being asked to disclose; for example, those who had been relatively recently diagnosed,^58^ who had experienced an acute stressor,^33,57^ or who had exhibited higher baseline levels of distress.^57,61,63^ However, others noted that short-term emotional disclosure-based interventions may not produce enduring effects due to the evolving nature of advanced illness, suggesting booster sessions as a possible solution.^75^ And others suggested that the increased patient contact for people at an advanced stage of illness may in fact increase retention compared to those at an earlier stage of disease.^65^

#### Ensuring a safe environment for disclosure

The importance of creating an environment where people feel comfortable to share difficult feelings was frequently highlighted.^25,53–55,75,78,80^ This related to the physical environment; for example, setting the intervention in a safe space, such as the participant’s home, or a private room.^25^ It also referred to contextual factors, such as incorporating other soothing or positive elements that facilitate feelings of comfort^53,55^ and healthcare professionals endorsing the intervention and framing it as safe and trustworthy.^54^ The importance of creating a safe environment extended to ensuring that family carers felt comfortable with the participant taking part in the intervention;^33,55^ this can be particularly salient in non-Western countries, such as China, where there are cultural barriers to expressing emotions.^53^ Some noted that partner-based interventions improved retention and feasibility over private interventions,^62,67^ suggesting the presence of a partner may contribute to feeling safe and supported. However, challenges associated with dyads were also reported, such as inhibitions around disclosing emotions to a partner or worry about burdening them.^62^

#### Flexibility of intervention

Flexibility in format and delivery was often noted as a facilitator. This is partially related to the variable health of participants and location of where people were receiving care; where expression sessions were held at structured times and places, participants were often not able to attend or complete the intervention due to illness or other appointments.^25,63^ Likewise, if the intervention was only delivered in a specific room at the hospice, it became less accessible for people who were unable to leave their home, or bed.^25,55,66^ The place where people feel most safe to disclose their emotions can also vary between individuals; thus it is important to provide flexibility about the intervention location.^25^ Likewise, authors noted that there were individual differences in the format with which people felt comfortable disclosing their emotions, related to factors such as stage of disease,^33,52^ differences in education, or simply personal preference.^25,54,66^

#### Clarity and structure of instructions

A number of authors commented on clarity of instructions as an important factor in ensuring adherence to the core expressive components of the intervention, particularly for self-directed interventions.^33,58,63,66^ In one study it was noted that despite instructions asking participants to focus on their feelings, the tendency was to describe a factual account of their illness journey, undermining the emotional expression objective of the intervention.^66^ Whilst a certain amount of structure and guidance on disclosure topics was highlighted as important, opportunity to move beyond the prompts and experience self-revelation was also highlighted as valuable.^53,63^ Another study highlighted that interventions with an unstructured format may be better suited to those with higher baseline emotional expressivity.^59^ It was also suggested that building in additional supportive components, such as coping skills training, may help to optimally manage distress.^59,75^

#### Staff engagement and training

The importance of staff endorsement to build trust, staff knowledge, and management support were noted as key for successful implementation.^54,55^ Providing staff with information about the intervention was also noted to help allay their fears around how to respond to patients bringing up emotional concerns.^54,69^ Others highlighted that when delivering the intervention in the palliative care unit or hospice, there were interruptions from staff, and that there could be difficulties in finding an appropriate space, which may require management support.^27,30^ Finally, one study noted the importance of clear communication during the consent process, as some participants declined taking part because they did not feel entitled to further treatment for their mental wellbeing, since they were already receiving holistic care from their hospice team.^35^

## Discussion

### Main findings

This scoping review developed a multi-level taxonomy, grouping emotional disclosure-based interventions for people with advanced disease into three operative domains: topic, format, and dose of disclosure. Within each domain, intervention characteristics were grouped into classes, and each class mapped to reported efficacy. An earlier systematic review already showed that the overall evidence of expressive writing efficacy is mixed.^24^ The present review unpicked a broader range of emotional disclosure-based interventions to determine if there is any indication of which characteristic, or combination of characteristics, may hold the most therapeutic potential. Whilst there were no clear patterns in terms of which intervention characteristics in any domain were most effective, it was possible to identify a framework of potential key characteristics to guide further research.

#### Objectives and outcome measures

The objectives of emotional disclosure-based interventions varied, and included improvement of quality of life, as well as psychological, physical, and existential wellbeing. Most studies described the improvement of some aspect of psychological wellbeing as a primary objective. Many, though, provided vague descriptions of objectives. A range of outcome measures were employed to evaluate intervention efficacy, and follow-up time-points also varied. This reflects the uncertainty within the emotional disclosure and psychological intervention literature as a whole, on how best to evaluate such interventions.^30,81^ That said, these are holistic interventions and thereby impact is likely to be broad in terms of benefit.

#### Theoretical mechanisms

Authors drew on a wide range of psychological and social theories to inform and explain emotional disclosure-based intervention development and effect; this is similar to other reviews.^29–31^ However, these were rarely fully developed into causal mechanisms. Medical Research Council guidelines suggest that effective intervention development should be based on a clear understanding of its causal mechanisms.^26,36^ As emotional disclosure-based interventions vary across a number of domains, a single, cohesive theoretical framework to fit all emotional disclosure-based interventions is unlikely to be suitable. Rather, when developing interventions, researchers should focus on proposing theoretical accounts to justify the intervention design. Some studies in this review harnessed the potential of qualitative or linguistic analysis of disclosure texts to explore underlying mechanisms;^25,55,76–79,80^ this represents a potentially fruitful direction for future research. Such theoretical work can in turn inform appropriate outcome measure selection. In line with the wider psychosocial intervention literature,^82^ findings highlight that there are likely to be individual differences in response to emotional disclosure. Clarifying the underlying mechanisms and individual differences in response to emotional disclosure-based intervention will ultimately help clinicians to decide which, if any, forms of emotional disclosure-based interventions are likely to work for which people.

#### Facilitators and barriers

The review identified five themes relating to facilitators of and barriers to emotional disclosure-based intervention implementation and efficacy: impact of disease stage; ensuring a safe environment; flexibility; clarity and structure of instructions; and staff engagement and training. When developing interventions for people with advanced disease, it is crucial to understand the specific environment where these interventions will be implemented, and to adjust them accordingly.^26,36^ Unless an intervention can be effectively implemented, it will not be effective on a wide scale. As such, it is recommended that future research developing emotional disclosure-based interventions for the palliative care setting should pay attention to the themes highlighted here, in combination with appropriate co-design work to develop practically implementable interventions.^83^

### Strengths and limitations

A systematic, six-stage process based on scoping review guidelines was undertaken to capture and map a broad body of literature. This review applied a pragmatic, novel approach (modified Intervention Component Analysis) to synthesize insights into intervention characteristics, evaluation approaches, theoretical frameworks, and implementation factors, including studies that used a range of study designs. By including studies that were not designed to assess efficacy (such as feasibility and pilot studies), it was also possible to capture information about acceptability and feasibility. However, this limited the possibility of drawing clear links between intervention characteristics and efficacy. Regardless of this decision, the heterogeneity of intervention objectives and outcome measures made efficacy synthesis challenging. In light of this, one core strength of the review was the capture and analysis of experience-based evidence. This provided important insights into key implementation factors that should be considered in the design of interventions, but which are often overlooked in more traditional evidence syntheses. As all papers were graded as “Good” or “Fair” quality, this lends a certain degree of credibility to this evidence. However, due caution should still be applied when considering its strength since much of this data is based on informal author reflections.

Studies evaluated interventions in a range of palliative settings and populations. This strengthens the generalizability and relevance of findings to palliative care services, which usually provide care for people with a range of diagnoses. However, whilst people living with advanced disease do share common experiences, some physical and psychological challenges are uniquely associated with specific conditions. Should researchers use insights from this review to inform intervention development, it would be important to consult with relevant stakeholders to ensure they address population and setting-specific factors on a more granular level. The majority of included studies were conducted in Western countries (US and UK). There can be significant cultural differences in the ways death, disease, and emotional expression are viewed.^15,84^ It is critical researchers consider this when interpreting or applying the results of this review in non-Western countries, or areas with multi-cultural populations.

Since emotional disclosure is a component of many formats of psychological therapy, there was sometimes a lack of clarity over what constitutes an emotional disclosure-based intervention. Despite employing a rigorous, discursive process to determine eligibility, some level of subjectivity about the selection of papers remained. However, the review was not designed to exhaustively capture every study that has ever been conducted in the field. Rather, it was designed to identify different types of intervention that could be classified as “emotional disclosure-based,” to systematically assess their characteristics and to identify the reasons why they may or may not be effective in palliative populations.

### What this review adds

This review maps the range of emotional disclosure-based interventions tested in people with advanced disease and proposes a multi-level taxonomy classifying their core characteristics. This is important as these low-cost interventions have therapeutic potential in palliative care settings. The review could help researchers adopt a common language to describe emotional disclosure-based interventions for people with advanced disease (and perhaps beyond) and inform design of future research, including systematic reviews and meta-analyses. This paper describes paths for researchers to move forward with the development of interventions that can be practically implemented, drawing on key facilitators and barriers. It also provides recommendations into promising avenues for future intervention evaluation to help guide selection of appropriate outcome measures. Additionally, the paper acts as an exemplar of a review approach that may be used to inform development and evaluation of complex, multi-component interventions where pre-existing evidence is mixed.

## Conclusion

Based on a systematic scoping of a diverse literature, this review has mapped and drawn links between emotional disclosure-based intervention characteristics, objectives, outcome measures, efficacy, and implementation factors. By drawing on the Intervention Component Analysis method, it was possible to integrate information not usually considered in traditional reviews of intervention efficacy. This has allowed the proposal of novel evidence-based recommendations for future research aiming to develop and evaluate emotional disclosure-based interventions in palliative populations.

## Supplemental Material

sj-docx-1-pmj-10.1177_02692163211013248 – Supplemental material for Emotional disclosure in palliative care: A scoping review of intervention characteristics and implementation factors Supplemental material, sj-docx-1-pmj-10.1177_02692163211013248 for Emotional disclosure in palliative care: A scoping review of intervention characteristics and implementation factors by Daisy McInnerney, Nuriye Kupeli, Paddy Stone, Kanthee Anantapong, Justin Chan, Kate Flemming, Nicholas Troop and Bridget Candy in Palliative Medicine

sj-docx-2-pmj-10.1177_02692163211013248 – Supplemental material for Emotional disclosure in palliative care: A scoping review of intervention characteristics and implementation factors Supplemental material, sj-docx-2-pmj-10.1177_02692163211013248 for Emotional disclosure in palliative care: A scoping review of intervention characteristics and implementation factors by Daisy McInnerney, Nuriye Kupeli, Paddy Stone, Kanthee Anantapong, Justin Chan, Kate Flemming, Nicholas Troop and Bridget Candy in Palliative Medicine

sj-docx-3-pmj-10.1177_02692163211013248 – Supplemental material for Emotional disclosure in palliative care: A scoping review of intervention characteristics and implementation factors Supplemental material, sj-docx-3-pmj-10.1177_02692163211013248 for Emotional disclosure in palliative care: A scoping review of intervention characteristics and implementation factors by Daisy McInnerney, Nuriye Kupeli, Paddy Stone, Kanthee Anantapong, Justin Chan, Kate Flemming, Nicholas Troop and Bridget Candy in Palliative Medicine

sj-docx-4-pmj-10.1177_02692163211013248 – Supplemental material for Emotional disclosure in palliative care: A scoping review of intervention characteristics and implementation factors Supplemental material, sj-docx-4-pmj-10.1177_02692163211013248 for Emotional disclosure in palliative care: A scoping review of intervention characteristics and implementation factors by Daisy McInnerney, Nuriye Kupeli, Paddy Stone, Kanthee Anantapong, Justin Chan, Kate Flemming, Nicholas Troop and Bridget Candy in Palliative Medicine

sj-docx-5-pmj-10.1177_02692163211013248 – Supplemental material for Emotional disclosure in palliative care: A scoping review of intervention characteristics and implementation factors Supplemental material, sj-docx-5-pmj-10.1177_02692163211013248 for Emotional disclosure in palliative care: A scoping review of intervention characteristics and implementation factors by Daisy McInnerney, Nuriye Kupeli, Paddy Stone, Kanthee Anantapong, Justin Chan, Kate Flemming, Nicholas Troop and Bridget Candy in Palliative Medicine

sj-docx-6-pmj-10.1177_02692163211013248 – Supplemental material for Emotional disclosure in palliative care: A scoping review of intervention characteristics and implementation factors Supplemental material, sj-docx-6-pmj-10.1177_02692163211013248 for Emotional disclosure in palliative care: A scoping review of intervention characteristics and implementation factors by Daisy McInnerney, Nuriye Kupeli, Paddy Stone, Kanthee Anantapong, Justin Chan, Kate Flemming, Nicholas Troop and Bridget Candy in Palliative Medicine
