# Supplementary material for: Emotional disclosure in palliative care: A scoping review of intervention characteristics and implementation factors
Source: Palliat Med. 2021 May 29;35(7):1323–43. doi: 10.1177/02692163211013248 (PMC8267079; doi:10.1177/02692163211013248)
Supplement: sj-docx-1-pmj-10.1177_02692163211013248 – Supplemental material for Emotional disclosure in palliative care: A scoping review of intervention characteristics and implementation factors [file sj-docx-1-pmj-10.1177_02692163211013248.docx]

**Supplementary File 1. Summary of changes from published protocol**

**Protocol source:** McInnerney D, Kupeli N, Stone P, Anantapong K, Chan J, Candy B. Emotional disclosure as a therapeutic intervention in palliative care: a scoping review protocol. BMJ Open. 2019 Aug 26;9(8):e031046. doi: 10.1136/bmjopen-2019-031046. PMID: 31455716; PMCID: PMC6720334.

| Change from published protocol | Rationale |
| --- | --- |
| Eligibility criteria |  |
| **Population:** in the original protocol we had planned to include studies of emotional disclosure-based interventions in people with palliative-stage disease and their family carers. However, after title and abstract screening we decided that studies in family carers would be excluded.  **Interventions:** in the original protocol we had planned to include art therapy, music therapy and dance therapy, life review and dignity therapies. However, after title and abstract screening we decided to exclude these therapy types in order  **Study types**: in the original protocol we had planned to include reviews, opinion pieces and commentaries however we decided to exclude these after screening | To manage the size and scope of the review, enable more systematic analysis, and thus keep the results more focused |
| **Definition of emotional-disclosure based:** in the original protocol we defined this as   - *“studies must use or make reference to a psychotherapeutic intervention that the authors state involves ‘emotional disclosure’ or involves a task that requires participants to express or communicate feelings or emotions as a core or critical element of the therapy and that aims to improve some aspect of patient or carer well-being”*   Through iterative discussion this was developed into:   - *“ED-based psychotherapeutic interventions were included, provided they:*  1. *Described the method of at least one task or exercise as part of the intervention that is designed to encourage or facilitate the disclosure, expression or discussion of emotions or feelings AND* 2. *Described emotional disclosure or expression of emotions as a key goal, rationale or functional mechanism of the intervention”* | To provide more clarity for the screening team over which studies should/should not be included, following confusion over emotional disclosure being a ‘core or critical element’ of the intervention |
| Analysis |  |
| In the original protocol we planned to synthesise the results using thematic analysis, but did not explicitly state that we would be using Intervention Component Analysis, which we used to guide the synthesis in the review | Whilst planning the most appropriate synthesis methods, we came across ICA as an appropriate, systematic method to meet the objectives of the review. |
| Quality appraisal | |
| We decided to appraise the quality of included studies using the Hawker tool although quality appraisal was not planned in the original protocol. | Quality appraisal is not mandated in scoping review methodology guidelines so we did not originally plan to include it. However, since we sought to make recommendations based on the findings reported in the included papers, we recognised that it would be valuable to have an understanding of the quality of the included studies, and as such selected the Hawker tool to quality appraise the included studies, as this tool is suitable for assessing various study designs. |
