# Supplementary material for: Emotional disclosure in palliative care: A scoping review of intervention characteristics and implementation factors
Source: Palliat Med. 2021 May 29;35(7):1323–43. doi: 10.1177/02692163211013248 (PMC8267079; doi:10.1177/02692163211013248)
Supplement: sj-docx-2-pmj-10.1177_02692163211013248 – Supplemental material for Emotional disclosure in palliative care: A scoping review of intervention characteristics and implementation factors [file sj-docx-2-pmj-10.1177_02692163211013248.docx]

**Supplementary File 2. Database search strategies**

**Ovid MEDLINE(R) and Epub Ahead of Print, In-Process & Other Non-Indexed Citations and Daily**

1. exp Emotions/
2. emotion*
3. feeling*
4. 1 OR 2 OR 3

*************

1. Palliative Care/
2. Terminal care/
3. (palliat* or terminal* or endstage or hospice* or metasta* or (end adj3 life) or (care adj3 dying) or ((advanced or late or last or end or final) adj3 (stage* or phase*))).tw.
4. 5 OR 6 OR 7

*********

1. (disclos* or express* or communicat* or talk* or speak* or spoke* or writ* or draw* or sing*).mp.

*********

1. 4 AND 8 AND 9

*********

1. Apply filter: Humans
2. Apply filter: All adults (19 plus years)

**Ovid PsycINFO**

1. exp Emotions/
2. emotion*
3. feeling*
4. 1 OR 2 OR 3

*************

1. Palliative Care/
2. (palliat* or terminal* or endstage or hospice* or metasta* or (end adj3 life) or (care adj3 dying) or ((advanced or late or last or end or final) adj3 (stage* or phase*))).tw.
3. 5 OR 6

*********

1. (disclos* or express* or communicat* or talk* or speak* or spoke* or writ* or draw* or sing*).mp.

*********

1. 4 AND 7 AND 8

*********

1. Apply filter: Humans
2. Apply filter: Adulthood (18+ years)

**Web of Science Core Collection**

#1 ALL FIELDS: (Emotion*)

#2 ALL FIELDS: (Feeling*)

#3 TOPIC: (((palliat* or terminal* or endstage or hospice* or metasta* or (end near/3 life) or (care near/3 dying) or ((advanced or late or last or end or final) near/3 (stage* or phase*)))))

#4 ALL FIELDS: (disclos* or express* or communicat* or talk* or speak* or spoke* or writ* or draw* or sing*)

#5 #2 or #1

#6 #5 AND #4 AND #3

**SCOPUS**

1 TITLE-ABS-KEY ( emotion* )

2 TITLE-ABS-KEY ( feeling* )

3 TITLE-ABS-KEY (( ( ( palliat*  OR  terminal*  OR  endstage  OR  hospice*  OR  metasta*  OR  ( end  W/3  life )  OR  ( care  W/3  dying )  OR  ( ( advanced  OR  late  OR  last  OR  end  OR  final )  W/3  ( stage*  OR  phase* ) ) ) ) ) )

4 TITLE-ABS-KEY (disclos* or express* or communicat* or talk* or speak* or spoke* or writ* or draw* or sing*)

5 #1 OR #2

6 #3 AND #4 AND #5

7 Limited to subject areas: “SOCI” OR “PSYC” OR “NURS” OR “MEDI”

8 Excluded subject areas: “Pharmacology”, “Agricultural and biological sciences”, “Computer science”, “Engineering”, “Business, management and accounting”, “Immunology and microbiology”, “Mathematics”, “Dentistry”, “Economics, econometrics and finance”, “Veterinary”, “Chemical engineering”, “Environmental science”, “Materials science”, “Chemistry”, “Physics and astronomy”

9 Exclude keywords: “Adolescent”, “Child” “Nonhuman”

10 Excluded document types: “Book”, “Book chapter”

11 Limited to language: “English”

**CINAHL Plus**

S1 MH “Emotions+”

S2 emotion* OR feeling*

S4 MH “Emotional support (IOWA NIC)”

S5 MH “Emotional support (SABA CCC)+”

S6 S1 OR S2 OR S3 OR S4 OR S5

S7 MH “Palliative Care”

S8 MH “Terminal Care+”

S9 palliat* or terminal* or endstage or hospice* or metasta* or (end N3 life) or (care N3 dying) or ((advanced or late or last or end or final) N3 (stage* or phase*))

S10 S7 OR S8 OR S9

S11 disclos* or express* or communicat* or talk* or speak* or spoke* or writ* or draw* or sing*

S12 S6 AND S10 AND S11

Limits applied: all adult

Limits applied: English

**CENTRAL**

#1 MeSH descriptor: [Emotions] explode all trees

#2 emotion* OR feeling*

#3 #1 OR #2

#4 MeSH descriptor: [Palliative Care] explode all trees

#5 MeSH descriptor: [Terminal Care] explode all trees

#6 palliat* or terminal* or endstage or hospice* or metasta*

#7 (end N3 life) or (care N3 dying) or ((advanced or late or last or end or final) N3 (stage* or phase*))

#8 #4 OR #5 OR #6 OR #7

#9 disclos* or express* or communicat* or talk* or speak* or spoke* or writ* or draw* or sing*

#10 #3 AND #8 AND #9

**OTHER SOURCES**

**The European Union clinical trials register**

- (emotions or feelings or emotional or emotionally) and (disclosure or disclose or expressive or expression or express or writing or drawing or expressing or disclosing) and (palliative care or terminal or hospice or advanced cancer or advanced disease or metastatic or end-of-life)

**clinicaltrials.gov**

- Terminal illness + emotional disclosure
- Terminal illness + expressive writing
- Terminal illness + disclosure
- Terminal illness + expression
- End Stage Disease + emotional disclosure
- End Stage Disease + expressive writing
- End Stage Disease + disclosure
- End Stage Disease + expression
- End Stage Disease + emotion

The European Association for Palliative Care (EAPC) conference abstract proceedings for the last 14 (2005-2019) – downloaded

British Psychological Society (BPS) conference abstract proceedings for the last 17 years (2001 – 2019) – downloaded
