# Supplementary material for: Emotional disclosure in palliative care: A scoping review of intervention characteristics and implementation factors
Source: Palliat Med. 2021 May 29;35(7):1323–43. doi: 10.1177/02692163211013248 (PMC8267079; doi:10.1177/02692163211013248)
Supplement: sj-docx-3-pmj-10.1177_02692163211013248 – Supplemental material for Emotional disclosure in palliative care: A scoping review of intervention characteristics and implementation factors [file sj-docx-3-pmj-10.1177_02692163211013248.docx]

**Supplementary File 3. Data extraction form**

| Reference #: | Reference |
| --- | --- |
| **Aims and type/mode of intervention:** | **Aim of study:**  **Aim of intervention:**  **Mode of intervention**:  **Mode of control/comparator:** |
| **Rationale, mechanism or theory:** |  |
| **Based on previous interventions?** |  |
| **Stakeholders consulted prior to development?** |  |
| **Population:** | Description (XX male/female with…)  **Mean age:**  **Ethnicity:** |
| **Who delivers and training:** | **Who:**  **Training:** |
| **Method of inviting disclosure:** |  |
| **Materials and method of disclosing:** | **Method:**  **Materials:**  **Location:** |
| **Topic of disclosure:** |  |
| **Dosing:** |  |
| **Does it work (inc. outcome measures)** | **Assessments (when):**  **Measures:**  **Results:**  **Other analyses:** |
| **Facilitators** |  |
| **Barriers** |  |
| **Delivery** |  |
| **Quality** |  |
| **Future research recommendations** |  |
| **Other notes of interest** |  |
