# Supplementary material for: Emotional disclosure in palliative care: A scoping review of intervention characteristics and implementation factors
Source: Palliat Med. 2021 May 29;35(7):1323–43. doi: 10.1177/02692163211013248 (PMC8267079; doi:10.1177/02692163211013248)
Supplement: sj-docx-4-pmj-10.1177_02692163211013248 – Supplemental material for Emotional disclosure in palliative care: A scoping review of intervention characteristics and implementation factors [file sj-docx-4-pmj-10.1177_02692163211013248.docx]

Supplementary file 4. Detailed study characteristics and reported efficacy of ED-based interventions in included studies

| Reference | Description of intervention | Outcome measures | Did it work* |
| --- | --- | --- | --- |
| RCTs |  |  |  |
| **Arden-Close et al. 2013**(1)  *102 women at* ***all stages of ovarian cancer (>50% at Stage III or IV )*** *and their partners recruited via ovarian cancer charity*  *UK* | **Expressive writing (for couples)** – based on Guided Disclosure Protocol (trauma) (GDP)  **Control** – neutral writing task (what they did the previous day) | *Primary*: PSS for perceived stress, FACT-G for QoL  *Secondary*: IES-R for intrusive thoughts, Couples Illness Communication Scale  *When*: Baseline, 3- and 6-month | **No main effects of GDP on stress / QoL outcomes** compared to control 3 or 6 months after the intervention  Change in illness-related communication moderated effect of group on QoL in patients: **GDP improved QoL if illness-related couple communication improved** (B=1.17, SE=0.52, b=0.20; F(1, 85) = 5.16, p=0.03) **at 3-month follow-up**  Change in intrusive thoughts moderated the effect of group on perceived stress in patients: **intrusive thoughts did not increase perceived stress in GDP group but did in control** (B =-0.43, SE=0.16, b -2.66; F(1, 84) = 7.07, p = 0.009) **at 3 month follow-up**  **No group by time interaction on intrusive thoughts or illness-related couple communication**.  **No qualitative results reported** |
| **Averill et al. 2013** (2)  *33 males and 15 females with* ***ALS likely to survive for at least 6 months*** *with good psychological health recruited via ALS registries*  *US* | **Written or spoken emotional disclosure**  **Control** – no disclosure exercise | *Primary:* Psychological wellbeing (primary) – composite measure of: ABS, GDS, psychological and existential QoL (McGill QoL questionnaire)  *Secondary:* AEE, emotional approach coping (eight item scale developed by Stanton et al. 2000), social constraints scale  *When:* Baseline, immediately post-intervention, 3- and 6-month | Statistically significant interaction between group and time predicting psychological well-being (F(2, 69) = 4.27, p < .05): **intervention improved psychological wellbeing (composite measure, -0.031 to 0.66, p<0.05) at 3 months**, but not 6 months post- intervention.  **Higher ambivalence was associated with a more robust response to the intervention at month 3 (p=0.05)**  **Mode of disclosure** had no effect on well-being as main effect or interaction with time  **No short term effects (negative or positive) of disclosure on negative affect (ABS)** immediately after disclosure compared to control  **No qualitative results reported** |
| **Bruera et al. 2008** (3)  *15 females/9 males with* ***advanced gynaecological and prostate cancer*** *referred to palliative care or inpatient unit*  *US* | **Expressive writing**  **Control –** neutral writing | *Primary:* STAI for anxiety  *Secondary:* BSI-18 for psychological distress, PSS for stress, ESAS for symptoms, FACIT-F for functional symptoms, PSQI for sleep  *When:* At baseline and STAI immediately pre and post intervention and at end of 2 week study period, all others at end of 2 weeks | The study concluded that **clinical trials of expressive writing in the palliative care setting are not feasible** unless they undergo major modification in methods as compared to other patient populations.  There was **no significant difference in the STAI State-Anxiety scores at baseline, before and after each writing session** between the EW and control group. Results for other measures not reported.  **No qualitative results reported** |
| **De Moor et al. 2002** (4)  *36 males and 6 females with* ***metastatic Stage IV Renal Cell Carcinoma*** *recruited from a Phase II tumour vaccine trial with life expectancy more than 4 months*  *US* | **Expressive writing**  **Control** – neutral writing | IES, PSS for stress; POMS for mood; PSQI for sleep disturbances  *When:* Baseline measures prior to randomisation, day of the fourth writing session and 4, 6, 8, and 10 weeks later. | Patients in the EW group reported **better sleep and better daytime functioning (PSQI)** than in the control group on total PSQI score (ES=0.73, p = .04) and 4 of the 5 sub-scales (mean scores across post-intervention FU time points)  **No significant difference between group means on distress or mood measures, except a significantly better score on the Vigor subscale of POMS in the EW group** (ES =0.82, p = .03).  **No qualitative results reported** |
| **Imrie, S. and N. A. Troop (2012)** (5)  *8 females and 5 males with* ***life-limiting illness or secondary cancer*** *recruited from a Day Hospice*  *UK* | **Compassion-focussed expressive writing (CFEW)**  **Control -** expressive writing about stress without compassion instruction (control) | FSCRS for self-soothing; SISE for self-esteem; SDHS for mood; PSS10 for stress; LIWC analysis of written essays  *When:* Baseline (week 1) and week 3 post-intervention | **Insufficient data was collected for inferential statistics**  **Writing tasks were well received and perceived as personal, meaningful and valuable – by all participants in both groups**  LIWC analysis found both groups reduced the number of negative words they used between baseline and follow-up (*F*1,18 = 6.97, *p* < 0.02) but compared to the control group, the expressive writers increased the number of causal words used over time (*F*1,18 = 8.36, *p* < 0.01)  **No qualitative results reported** |
| **Lloyd Williams et al. 2013** (6)  *68 females and 32 males with* ***advanced metastatic cancer (range of primary sites)*** *at the end stage of their diseases recruited from hospice day units.*  *UK* | Spoken disclosure as part of a **focussed narrative interview.**  **Control** – usual care | *Primary:* Numerical Visual Analogue Scale of global suffering  *Secondary:* BEDS for depression (6 item); FACIT-Sp (spirituality); ESAS for pain, tiredness, nausea, depression, anxiety, drowsiness, appetite, wellbeing, breathlessness, + ‘will to live’ item); ECOG for performance status  *When:* At baseline, 2, 4 and 8 weeks post-intervention | **Significant improvement in pain** (ESAS) at 8 weeks (p<0.01) compared to control  **No significant improvement in any other measure** at any time point between IG and control (usual care)  **No qualitative results reported** |
| **Lloyd Williams et al. 2018** (7)  *39 females and 18 males with* ***advanced cancer receiving palliative care*** *from a hospice day care service with a prognosis between 6 weeks and 12 months, with clinical depression*  **UK** | Spoken disclosure as part of a **focussed narrative semi-structured interview.**  **Control** – usual care | *Primary: PHQ-9 for depression*  *Secondary:* Single-item PROM for depression, ESAS symptom assessment  *When:* Baseline, and 2, 4 and 6 weeks post-intervention | **Significantly more patients in the intervention group had a clinically relevant reduction in depression score (PHQ-9)** at 6 week follow-up (11 vs 2; p=0.04) compared to control (usual care).  **There was no significant difference in clinically significant depression score reduction at week 2 or week 4 follow-up.**  Changes in ESAS scores not reported  **No qualitative results reported** |
| **Low et al. 2010** (8)  *62 women with stage* ***IV metastatic breast cancer*** *receiving any form of treatment, recruited from larger study from oncology clinics, community practices and online mBC website*  *US* | **Expressive writing**  **Control** – writing about facts of cancer diagnosis and treatment | CES-D for depression, intrusions subscale of IES, negative somatic symptoms - using a measure shown to be responsive to EW developed by Pennebaker (1982; Stanton et al., 2002)); PSQI for sleep  *When:* Baseline and 3 months post-intervention | **No significant main effects** of EW were observed on depressive symptoms, cancer-related intrusive thoughts, somatic symptoms or sleep quality at 3 months post-intervention  Significant condition × social support interaction on intrusive thoughts: **EW was associated with reduced intrusive thoughts for women reporting low emotional support (η2 = .15, p=0.02).**  Significant condition × time since metastatic diagnosis on somatic symptoms and sleep disturbances: **EW participants who were more recently diagnosed had fewer somatic symptoms (η2 = .10, p=0.034)**, whereas **EMO participants with longer diagnosis duration exhibited increases in sleep disturbances** (η2 = .09, p=0.023).  **No qualitative results reported** |
| **Manne et al. 2007** (9)  *253 women with* ***gynaecological cancer (>50% advanced)*** *recruited from cancer centres and hospitals*  *US* | **Supportive counselling**  **Comparator** – Coping and Communication Intervention (CCI)  **Control** – Usual care | BDI for depressive symptoms; IES for cancer-specific distress  *When:* Baseline, 3, 6 and 9 months post-intervention | **There was no significant effect of group on depressive symptoms over time. At 9 month follow-up, Tukey-adjusted mean BDI depressive symptoms were significantly in SC group compared to UC** (t(339) = –2.40, p = .0443). There was no significant difference in means between SC and CCI.  This effect was stronger for women with temporal reductions in ECOG status and higher baseline levels of emotional expressivity.  Neither SC not CCI had any significant effect on IES at any timepoint, nor were any moderator interactions significant.  **No qualitative results reported** |
| **Manne et al. 2017**(10)**; Manne et al. 2017b** (11)**; Virtue et al.**  **2019** (12)**; Virtue et al.2015** (13)  *252 women with* ***gynaecological cancer (>50% advanced)*** *recruited from cancer centres and hospitals*  *US* | **Supportive counselling**  **Comparator** – Coping and Communication Intervention (CCI)  **Control** – Usual care | BDI for depressive symptoms; IES for cancer related distress; FACT-F (Emotional functioning subscale) for emotional functioning/QoL, and 4 item subscale of Concerns About Recurrence Scale adapted for gynaecological cancer  *When:* Baseline, 5 weeks, 9 weeks, 6 months, 12 months and 18 months post-baseline | **There were no significant differences at any time point on any measure as a function of condition comparing SC to UC or CCI.**  Positive emotional expressivity moderated cancer-specific distress (F (2319) = 6.47, p = 0.002), fear of recurrence (F (2323) = 11.39, p < 0.001) and emotional well-being (F(2, 321) = 6.78, p =0.001), driven by significant correlation between positive emotional expressivity and average outcome in the SC condition.  **No qualitative results reported** |
| **Milbury et al. 2018; 2019** (14,15)  *38 women and 37 men with* ***metastatic lung cancer*** *(and their partners) recruited from a cancer centre*  *US* | **Online couple-based meditation (CBM) with spoken emotional disclosure**  **Active control –** discussion of cancer related concerns in couples; no coping skills or probing for emotional disclosure  **Usual care waitlist control (WLC)** | Feasibility measures  *QoL measures:* FACT-SP for spiritual wellbeing; CES-D for depression; PSQI for sleep; MDASI-LC for QoL  *Process measures:* Mindful awareness scale; Self Compassion Scale; Pistrang and Barker’s Holding Back scale; Personal Assessment of Intimacy in Relationships instrument and Experiences in Closer Relationship Scale-Short Form.  When: Baseline, 4 weeks later, and 3 months later. | Intervention deemed **feasible and acceptable.**  Compared with the waitlist control group, patients in the CBM group reported significantly lower depressive symptoms (P = .02; d = .49; CES-D means: CBM = 7.87; SE = 11.51; WLC = 12.76) and cancer specific distress (P = .05; d = .44; IES means: CBM = 12.40; SE = 16.34; WLC = 18.22).  There were no significant effects between active and waitlist control groups.  **No qualitative results reported** |
| **Milbury et al. 2020** (16)  *16 women and 18 men with* ***primary or metastatic brain tumours (>50% advanced)*** *recruited from clinics*  *US* | **Online couple-based meditation (CBM) with spoken emotional disclosure**  **Control** – usual care | MDASI for cancer-specific symptoms; MAAS for mindfulness; SCS for self-compassion; PAIRI (six items) for intimacy; CES-D for depressive symptoms  When: Baseline, 6 and 12 weeks post-baseline (2 and 8 weeks post-intervention) | The results revealed that the trial and **CBM intervention was feasible** and met a priori feasibility criteria regarding consent, retention, and adherence rates.  Controlling for baseline levels, statistically **significantly improved cognitive** (F = 4.84; P < 0.05; d = 1.05) and **general disease** (F = 4.65; P < 0.05; d = 0.93) symptoms, **intimacy** (F = 7.63; P < 0.05; d = 0.68) **and self-compassion** (F = 5.98; P = 0.03; d = 0.96) in the CBM vs control group.  Improved depressive symptoms were reported in CMB group compared to control as “marginally significant” (F =3.91; P = 0.06; d = 0.70) but did not meet a priori significance level (5%).  **No qualitative results reported** |
| **Mosher et al. 2012** (17)  *87 women with* ***metastatic (Stage IV) breast cancer*** *attending comprehensive cancer centre with clinically elevated distress*  *US* | **Expressive writing**  **Control –** neutral writing | FACT-Sp (and an additional measure of demoralisation/existential despair/distress) for existential wellbeing; Distress Thermometer, CES-D, and HADS-A for psychological wellbeing; PSQI, FACT-F for sleep disturbance and fatigue, use of mental health services measure, LIWC analysis of essays, researcher and participant essay ratings  *When:* Baseline and ~ 8 weeks post-intervention | **No improvement in existential or psychological wellbeing, sleep quality or fatigue (measures)** at 8-weeks post intervention compared to control  **Significantly greater uptake of mental health services** in intervention group compared to neutral writing control (24/44 vs. 11/42, respectively; OR = 3.40, 95% CI, 1.05 to 11.08)  **No qualitative results reported** |
| **Porter, L. et al. (2009)** (18)  *92 males and 38 females with* ***gastrointestinal cancer (>50% advanced)*** *(and their partners) recruited from hospital oncology clinics*  *US* | **Partner-assisted spoken disclosure**  **Control** – 4x weekly education/support sessions | QMI for Relationship quality; MSIS for intimacy; POMS for psychological distress.  *When:* Baseline and after the intervention (time not specified) | **Patient-rated relationship quality significantly improved** pre to post treatment relative to control in the intention-to-treat (ITT) analysis (B=−.07, SE=0.03, p=.02) and treatment completers analysis ((B=−0.08, SE=0.04, p=.02). The **intervention improved intimacy** relative to control in the treatment completer’s analysis (B=−0.60, SE=0.30, p=.05), but this was only significant in the ITT analysis when patients reported high baseline levels of holding back (B=.56, SE=0.28, p=.02).  There were **no significant effects on psychological distress**.  **No qualitative results reported** |
| **Steinhauser, K. E., et al. (2008; 2009)** (19,20)  *38 female and 44 male hospice patients with* ***varying diagnoses and a prognosis of less than 6 months*** *to live, recruited from inpatient and outpatient hospital,*  *palliative care, and hospice settings*  *18 took part in qualitative interviews (2009).*  *US* | **Spoken disclosure (Outlook intervention)** in semi-structured, audio-recorded interview based on principles of emotional disclosure and life review.  **Control** – Relaxation Meditation | MSAS for pain and symptoms; QUAL-E for quality of life at the end of life; Rosow-Breslau ADL Scale for functional status; POMS anxiety sub-scale, CES-D short version for depression; Daily Spiritual Experience Scale; brief measure of family social support*  *To reduce burden, daily spiritual experience and family social support measures were not completed week 1 and 2 post-intervention.  *When:* Baseline, 5 and 7 weeks post-baseline (which is 1 and 3 weeks post-intervention) | **No significant differences over time in life completion or preparation, overall QOL, anxiety, or depression** compared to RM control at week 5 or 7 post-baseline  Qualitative analysis of interviews showed both positive and negative experiences were mentioned, and covered life roles, values, and accomplishments (session 1); choices one might have made differently, exploration of forgiveness offered and sought (session 2); lessons learned, heritage and legacy (session 3). |
| **Steinhauser, K. E., et al. (2017)** (21)  *212 male and 9 female* ***hospice ineligible advanced disease patients*** *(to understand benefits in early palliative care context) recruited from outpatient clinics*  *US* | **Spoken disclosure (Outlook intervention)** – as above – to improve patient QOL, functional status, and emotional well-being  **Control (1)** – Relaxation Mediation  **Control (2)** – Usual Care | *Primary:* QUAL-E - preparation and completion sub-scale  *Secondary:* FACT-G, POMS anxiety scale, CES-D for depression; FACT-Sp for spiritual wellbeing  *When:* Baseline, 5 and 7 weeks post-baseline (which is 1 and 3 weeks post-intervention)  Qualitative interviews assessed outlook intervention acceptability in ~15% participants (n=12) and content analysed for common themes using descriptive content analysis | **No significant differences over time in life completion or preparation, overall QOL, anxiety, or depression** compared to RM at week 5 or 7 post-baseline  Compared to UC, **higher improvements in mean preparation** (1.1; 95% CI 0.2, 2.0; P = 0.02) **and mean completion** (1.6; 95% CI 0.05, 3.1; P = 0.04) at five-week follow-up; these differences did not persist at the seven-week follow-up  **Qualitative findings:**  “Overwhelmingly” described intervention as helpful; nothing was reported as unhelpful. In general, respondents indicated that outlook let them relate as a whole person: “More about myself than my condition, which made me feel real good. The first time I talked about myself.”  4 major themes identified from interviews: memories as a source of strength, releasing difficult emotions, forgiveness, and a tool for coping. |
| **Zhu, J. et al (2019)** (22)  *6 males and 10 females with* ***incurable cancer*** *recruited from cancer clinics*  *US* | **Creative writing workshops (CWW) fostering emotional expression**  **Control** – standard of care | *Primary* – ETS for mental health  *When* – Before and after each CWW | The study concluded it is feasible for patients with cancer to attend focused CWW workshops intended to promote mental health. Seven out of 11 patients (63%) enrolled in IA attended at least 75% of classes  There was no significant difference between intervention and control groups on the ETS, except on the anxiety sub-scale, although there was a trend towards mood improvement relative to SOC when comparing initial and final ETS score.  In the CWW group, anxiety (−1.47, p=0.0012), depression (−1.01, p=0.0051), anger (−0.37, p=0.0251) and overall (-4.31, p=0.0018) ETS scores declined significantly overall between preclass and postclass. A significant decreasing trend was also observed for the total preclass ETS (average score decreases 1.43 per visit, p=0.0378).  **No qualitative results reported** |
| **Secondary analyses of RCTs** | | | |
| **Laccetti et al. 2007** (23)  *Descriptive, correlational secondary analysis of RCT*  *68 women with* ***metastatic breast cancer and life expectancy >6 months*** *recruited from medical centres, community centres and private clinic*  *US* | **Written disclosure** intervention to enhance quality of life and relieve symptoms | FACT-B for QoL at baseline and three months post-intervention  LIWC analysis of written narratives | LIWC analysis found a **significant relationship between positive-affect word use and emotional well-being subscale and the additional concerns subscale** (FACT-B) measured 3 months after intervention controlling for baseline scores.  **No qualitative results reported** |
| **Leal et al. 2018** (24)  *Qualitative evaluation of EW texts from RCT*  *16 females and 21 males with* ***renal cell carcinoma recruited*** *from RCT of EW in people with renal cell carcinoma of all stages*  *US* | **Written disclosure** intervention to reduce cancer related symptoms and improve physical functioning (QoL) | Qualitative analysis of written narratives | **Did not examine differences in outcomes.**  Main themes identified:   - Interconnection was the over-arching theme, with narratives describing a non-linear, ongoing and dynamic transition through discontinuity and loss following diagnosis, reorientation to a new reality, rebuilding a new life and expansion/altruistic contribution to something larger than oneself. - Distinction between developing and forcing positivity: avoiding pain exacerbated distress, as emotional avoidance curtails articulation, and processing. |
| **Rose et al. 2008; 2009; Radziewicz, 2009** (25–27)  *110 males and 51 females with* ***advanced cancer*** *(median life expectancy of one year or less) recruited from two ambulatory cancer clinics*  *US* | **Spoken disclosure** via telephone-based Coping and Communication Support (CCS) intervention | Assessed fidelity and group differences in engagement. | **Did not report efficacy of intervention** |
| **Non-RCTs** |  |  |  |
| **Garcia Perez, A. I. and J. J. Dapueto (2014)** (28)  *Case study*  *Female with* ***advanced ALS***  *Uruguay* | **Spoken disclosure in computer-assisted psychotherapy** | Case report | **The intervention resulted in better symptom control, improved communication with the team and family, reduction of psychological distress, promotion of autonomy, dignity, and self-esteem.** |
| **Milbury et al. 2018** (29)  *5 women and 8 men with primary or metastatic non-small cell lung cancer recruited from clinics*  *US* | **Couple-based meditation with spoken emotional disclosure** | Single arm trial (n=7) and intervention content evaluation sessions (n=7) (semi-structured interviews and written evaluations)  Measures for trial: CES-D for depression; PSQI for sleep; IES and FACT-Sp  When: Baseline and post-programme | Intervention content evaluation: High acceptability (all participants would recommend the intervention). Only suggested improvement was one request for more reading materials.  Single arm trial:   - “Acceptable” consent (54%) and adherence (67%) rates. All patients rated the intervention as useful (38%) or very useful (62%), and 75% indicted that they benefited greatly from it. - Positive open-ended comments: “*Was the most relaxed while awake I’ve been in a good while*,” ”*Excellent session - good opportunity to reflect on type of person I am and would like to be,*” “*Very good session. Understanding gratitude is very very important. Thank you for bringing our attention to this important attitude*,” and “*It really brought us closer and taught us to have more purpose with each other*.” - Paired t-test analyses revealed large effect sizes for reduced sleep disturbances (d = 1.83) and medium effect sizes for cancer-specific distress (d = 0.61) for patients but only significant for sleep disturbances. |
| **Pon et al. 2010** (30)  *Case studies*  *5 hospice patients with* ***terminal stage cancer with <6 months to live*** *recruited from a hospice programme*  *China* | **Spoken disclosure** in context of playing **‘My Wonderful Life’(MWL)** **board game** – participant moves along game board performing acts or picking an ‘Honest expression’ card | Pre-, post- and follow-up  interviews with the participants | **Positive qualitative feedback reported, including:**   1. Leaving a (non-materialistic) legacy 2. Preparing family and self for end of life; 3. Appreciating self and achievements; 4. Sharing of feelings, appreciation, and concerns as a release 5. Distractions from pain and negative thoughts 6. Facilitated patients entering into therapy and enhanced therapeutic outcomes (exploration and revelation were both sped up and deepened). 7. The game also offered a sense of closure   At the beginning of each MWL game, patients tended to be anxious and sad but, gradually these gave way to liberation, satisfaction, and fulfilment. |
| **Taylor et al. 2016** (31)  *Qualitative evaluation*  *24 male and 12 female patients with* ***end-stage renal disease*** *recruited from routine outpatient clinic*  *UK* | **Spoken disclosure in response to either:**   1. **Patient Issues Sheet (n=21) for participants to circle 2-3 main issues to discuss during consultation** (Intervention 1) or 2. **Direct well-being question adapted from PHQ-9 (n=20)** (Intervention 2) | Thematic analysis of semi-structured interviews with consultants and patients | **Both** **interventions were feasible and well-received.** All bar one participant thought clinicians should continue using the interventions, although a minority disliked the question as feared stigma or relevance to their current mood.  Five themes identified:  1. Patient and consultants adapted interventions adapted to personal style  2. Patients were enabled to raise emotional issues (patients) – felt cared for and allowed to use time to discuss emotions  3. Consultants were facilitated to explore emotional issues  4. Handling discussion (patients)  5. Training was valued by consultants |
| **Tuck et al. 2012** (32)  *Mixed methods*  *4 male and 3 females with a* ***diagnosis of terminal cancer*** *recruited from palliative care unit*  *US* | **Narrative storytelling (spoken, audio-recorded and transcribed) through the** PATS (Presence, Active Listening, Touch, Sacred story) intervention to respond to spiritual needs/healing and positively impact patient wellbeing. | SHI at study admission and 5 days later  Thematic analysis of transcribed PATS interviews, and open-ended evaluative questions  about the intervention | PATS was deemed **partially acceptable and feasible** with modifications – the **storytelling process was acceptable and feasible**  2^nd^ administration of SHI (5 days after intervention) was only completed in 3 patients therefore it was not possible to assess effect on spiritual wellbeing, but authors note results indicate potential for a ‘modest, positive effect’ on participant wellbeing.  Narrative analysis of transcripts revealed participants disclosed along 3 main themes:   1. Finding out and struggling 2. Discovering what it means 3. Living with the prognosis   Strong spiritual references were identified in the transcripts |

**We describe results as effective based on the analysis and statistical significance reported in the paper, although we recognise that this is limited in that it provides no indication of the suitability of analysis technique used or reliability of the reported analysis.*

**References**

1. Arden-Close E, Gidron Y, Bayne L, Moss-Morris R. Written emotional disclosure for women with ovarian cancer and their partners: randomised controlled trial. Psychooncology. 2013 Oct;22(10):2262–9.

2. Averill AJ, Kasarskis EJ, Segerstrom SC. Expressive disclosure to improve well-being in patients with amyotrophic lateral sclerosis: a randomised, controlled trial. Psychol Health. 2013;28(6):701–13.

3. Bruera E, Willey J, Cohen M, Palmer JL. Expressive writing in patients receiving palliative care: a feasibility study. J Palliat Med. 2008 Feb;11(1):15–9.

4. de Moor C, Sterner J, Hall M, Warneke C, Gilani Z, Amato R, et al. A pilot study of the effects of expressive writing on psychological and behavioral adjustment in patients enrolled in a Phase II trial of vaccine therapy for metastatic renal cell carcinoma. Health Psychology. 2002;21(6):615–9.

5. Imrie S, Troop NA. A pilot study on the effects and feasibility of compassion-focused expressive writing in Day Hospice patients. Palliat Support Care. 2012 Jun;10(2):115–22.

6. Lloyd-Williams M, Cobb M, O’Connor C, Dunn L, Shiels C. A pilot randomised controlled trial to reduce suffering and emotional distress in patients with advanced cancer. J Affect Disord. 2013 May 15;148(1):141–5.

7. Lloyd-Williams M, Shiels C, Ellis J, Abba K, Gaynor E, Wilson K, et al. Pilot randomised controlled trial of focused narrative intervention for moderate to severe depression in palliative care patients: DISCERN trial. Palliative Medicine. 2018 Jan;32(1):206–15.

8. Low CA, Stanton AL, Bower JE, Gyllenhammer L. A randomized controlled trial of emotionally expressive writing for women with metastatic breast cancer. Health Psychol. 2010 Jul;29(4):460–6.

9. Manne S, Rubin S, Edelson M, Rosenblum N, Bergman C, Hernandez E, et al. Coping and communication-enhancing intervention versus supportive counseling for women diagnosed with gynecological cancers. - PsycNET. Journal of Consulting and Clinical Psychology. 2007;75(4):615–628.

10. Manne SL, Virtue SM, Ozga M, Kashy D, Heckman C, Kissane D, et al. A Comparison of Two Psychological Interventions for Newly-diagnosed Gynecological Cancer Patients. Gynecol Oncol. 2017 Feb;144(2):354–62.

11. Manne SL, Myers-Virtue S, Darabos K, Ozga M, Heckman C, Kissane D, et al. Emotional processing during psychotherapy among women newly diagnosed with a gynecological cancer. Palliat Support Care. 2017;15(4):405–16.

12. Virtue SM, Manne S, Criswell K, Kissane D, Heckman C, Rotter D. Levels of emotional awareness during psychotherapy among gynecologic cancer patients [Internet]. Palliative & supportive care. 2019 [cited 2020 Jul 20]. Available from: https://pubmed.ncbi.nlm.nih.gov/29880065/

13. Myers Virtue S, Manne SL, Darabos K, Heckman CJ, Ozga M, Kissane D, et al. Emotion episodes during psychotherapy sessions among women newly diagnosed with gynecological cancers: Emotion episodes during psychotherapy with cancer patients. Psycho-Oncology. 2015 Sep;24(9):1189–96.

14. Milbury K, Li, Y, Durrani S, Liao Z, Yang C, Tsao A, et al. Results of a pilot randomized controlled trial: A couple-based meditation intervention for patients with metastatic lung cancer and their partners. | Journal of Clinical Oncology. Journal of Clinical Oncology. 2019;37(31_suppl):135–135.

15. Milbury K, Tsao AS, Liao Z, Owns A, Engle R, Gonzalez EA, et al. A research protocol for a pilot randomized controlled trial designed to examine the feasibility of a couple-based mind-body intervention for patients with metastatic lung cancer and their partners. Pilot and Feasibility Studies. 2018 Jan 24;4(1):37.

16. Milbury K, Weathers Spw, Durrani S, Li Y, Whisenant M, J L, et al. Online Couple-Based Meditation Intervention for Patients With Primary or Metastatic Brain Tumors and Their Partners: Results of a Pilot Randomized Controlled Trial [Internet]. Journal of pain and symptom management. 2020 [cited 2020 Jul 20]. Available from: https://pubmed.ncbi.nlm.nih.gov/32061834/

17. Mosher CE, Duhamel KN, Lam J, Dickler M, Li Y, Massie MJ, et al. Randomised trial of expressive writing for distressed metastatic breast cancer patients. Psychol Health. 2012;27(1):88–100.

18. Porter LS, Keefe FJ, Baucom DH, Hurwitz H, Moser B, Patterson E, et al. Partner-Assisted Emotional Disclosure for Patients with GI Cancer: Results from a Randomized Controlled Trial. Cancer. 2009 Sep 15;115(18 Suppl):4326–38.

19. Steinhauser KE, Alexander SC, Byock IR, George LK, Olsen MK, Tulsky JA. Do preparation and life completion discussions improve functioning and quality of life in seriously ill patients? Pilot randomized control trial. J Palliat Med. 2008 Nov;11(9):1234–40.

20. Steinhauser KE, Alexander SC, Byock IR, George LK, Tulsky JA. Seriously ill patients’ discussions of preparation and life completion: an intervention to assist with transition at the end of life. Palliat Support Care. 2009 Dec;7(4):393–404.

21. Steinhauser KE, Alexander S, Olsen MK, Stechuchak KM, Zervakis J, Ammarell N, et al. Addressing Patient Emotional and Existential Needs During Serious Illness: Results of the Outlook Randomized Controlled Trial. J Pain Symptom Manage. 2017;54(6):898–908.

22. Zhu J, Hussain M, Joshi A, Truica CI, Nesterova D, Collins J, et al. Effect of creative writing on mood in patients with cancer. BMJ supportive & palliative care. 2020 Mar 1;10(1):64–7.

23. Laccetti M. Expressive writing in women with advanced breast cancer. Oncol Nurs Forum. 2007 Sep;34(5):1019–24.

24. Leal I, Milbury K, Engebretson J, Matin S, Jonasch E, Tannir N, et al. Interconnection: A qualitative analysis of adjusting to living with renal cell carcinoma. Palliat Support Care. 2018;16(2):146–54.

25. Rose JH, Bowman KF, Radziewicz RM, Lewis SA, O’Toole EE. Predictors of Engagement in a Coping and Communication Support Intervention for Older Patients with Advanced Cancer: Patient engagement in a coping and communication support intervention. Journal of the American Geriatrics Society. 2009 Nov;57:s296–9.

26. Rose JH, Radziewicz R, Bowmans KF, O’Toole EE. A coping and communication support intervention tailored to older patients diagnosed with late-stage cancer. Clin Interv Aging. 2008;3(1):77–95.

27. Radziewicz RM, Rose JH, Bowman KF, Berila RA, O’Toole EE, Given B. Establishing treatment fidelity in a coping and communication support telephone intervention for aging patients with advanced cancer and their family caregivers. Cancer Nurs. 2009 Jun;32(3):193–202.

28. García Pérez AI, Dapueto JJ. Case report of a computer-assisted psychotherapy of a patient with ALS. Int J Psychiatry Med. 2014;48(3):229–33.

29. Milbury K, Engle R, Tsao A, Liao Z, Owens A, Chaoul A, et al. Pilot Testing of a Brief Couple-Based Mind-Body Intervention for Patients With Metastatic Non-Small Cell Lung Cancer and Their Partners. Journal of Pain and Symptom Management. 2018 Mar 1;55(3):953–61.

30. Pon AKL. My Wonderful Life: A Board Game for Patients with Advanced Cancer. Illness, Crisis & Loss. 2010 Apr;18(2):147–61.

31. Taylor F, Combes G, Hare J. Improving clinical skills to support the emotional and psychological well-being of patients with end-stage renal disease: a qualitative evaluation of two interventions. Clin Kidney J. 2016 Jun;9(3):516–24.

32. Tuck I, Johnson SC, Kuznetsova MI, McCrocklin C, Baxter M, Bennington LK. Sacred healing stories told at the end of life. J Holist Nurs. 2012 Jun;30(2):69–80.
