# Supplementary material for: Emotional disclosure in palliative care: A scoping review of intervention characteristics and implementation factors
Source: Palliat Med. 2021 May 29;35(7):1323–43. doi: 10.1177/02692163211013248 (PMC8267079; doi:10.1177/02692163211013248)
Supplement: sj-docx-5-pmj-10.1177_02692163211013248 – Supplemental material for Emotional disclosure in palliative care: A scoping review of intervention characteristics and implementation factors [file sj-docx-5-pmj-10.1177_02692163211013248.docx]

**Supplementary File 5. Quality appraisal results using Hawker tool**

| **Paper** | **Scoring** | | | | | | | | | | | **Overall rating** |
| --- | --- | --- | --- | --- | --- | --- | --- | --- | --- | --- | --- | --- |
|  | Study design | Abstract and title | Introduction and aims | Method and data | Sampling | Data analysis | Ethics and bias | Results | Transferability or generalisability | Implications and usefulness | Implications and usefulness: missing |  |
| Arden-Close, et al. (2013). (1) | RCT | Good (4) | Good (4) | Good (4) | Fair (3) | Good (4) | Fair (3) | Good (4) | Fair (3) | Fair (3) | Policy or practice implications | Good (32) |
| Averill, et al. (2013). (2) | RCT | Fair (3) | Good (4) | Good (4) | Poor (2) | Good (4) | Poor (2) | Good (4) | Poor (2) | Good (4) |  | Good (29) |
| Bruera, et al. (2008). (3) | RCT | Good (4) | Poor (2) | Poor (2) | Poor (2) | Poor (2) | Poor (2) | Poor (2) | Poor (2) | Good (4) |  | Fair (22) |
| de Moor, et al. (2002). (4) | RCT | Good (4) | Good (4) | Fair (3) | Fair (3) | Fair (3) | Poor (2) | Fair (3) | Fair (3) | Fair (3) | Policy or practice implications | Good (28) |
| Imrie and Troop (2012). (5) | RCT | Good (4) | Poor (2) | Good (4) | Poor (2) | Fair (3) | Good (4) | Fair (3) | Poor (2) | Good (4) |  | Good (28) |
| Lloyd-Williams, et al. (2013). (6) | RCT | Fair (3) | Poor (2) | Fair (3) | Poor (2) | Fair (3) | Poor (2) | Fair (3) | Fair (3) | Fair (3) | Policy or practice implications | Fair (24) |
| Lloyd-Williams, et al. (2018). (7) | RCT | Good (4) | Good (4) | Good (4) | Fair (3) | Good (4) | Good (4) | Good (4) | Good (4) | Good (4) |  | Good (35) |
| Low, et al. (2010). (8) | RCT | Fair (3) | Good (4) | Good (4) | Good (4) | Good (4) | Poor (2) | Good (4) | Good (4) | Good (4) |  | Good (33) |
| Manne, et al. 2007 (9) | RCT | Fair (3) | Fair (3) | Good (4) | Fair (3) | Good (4) | Fair (3) | Fair (3) | Fair (3) | Good (4) |  | Good (30) |
| Manne et al. (2017). (10) | RCT | Good (4) | Fair (3) | Good (4) | Poor (2) | Good (4) | Fair (3) | Good (4) | Poor (2) | Good (4) |  | Good (30) |
| Manne et al. (2017b). (11) | Analysis of RCT | Fair (3) | Good (4) | Good (4) | Good (4) | Good (4) | Good (4) | Good (4) | Fair (3) | Fair (3) | Further research | Good (33) |
| Virtue et al. (2019). (12) | Analysis of RCT | Fair (3) | Good (4) | Fair (3) | Fair (3) | Good (4) | Fair (3) | Good (4) | Fair (3) | Good (4) |  | Good (31) |
| Virtue et al. (2015). (13) | Analysis of RCT | Fair (3) | Good (4) | Good (4) | Fair (3) | Good (4) | Fair (3) | Good (4) | Fair (3) | Good (4) |  | Good (32) |
| Milbury, et al. (2020). (14) | RCT | Good (4) | Good (4) | Good (4) | Good (4) | Fair (3) | Fair (3) | Good (4) | Good (4) | Good (4) |  | Good (34) |
| Mosher, et al. (2012). (15) | RCT | Fair (3) | Good (4) | Good (4) | Fair (3) | Good (4) | Poor (2) | Good (4) | Good (4) | Good (4) |  | Good (32) |
| Porter, et al. 2009 (16) | RCT | Good (4) | Good (4) | Fair (3) | Poor (2) | Good (4) | Fair (3) | Fair (3) | Poor (2) | Good (4) |  | Good (29) |
| Steinhauser, et al. (2008). (17) | RCT | Fair (3) | Poor (2) | Fair (3) | Poor (2) | Poor (2) | Poor (2) | Fair (3) | Poor (2) | Good (4) |  | Fair (23) |
| Steinhauser, et al. (2009). (18) | Qualitative analysis of RCT | Good (4) | Poor (2) | Fair (3) | Fair (3) | Fair (3) | Fair (3) | Fair (3) | Fair (3) | Good (4) |  | Good (28) |
| Steinhauser, K. E., et al. (2017). (19) | RCT | Good (4) | Good (4) | Good (4) | Good (4) | Fair (3) | Good (4) | Good (4) | Fair (3) | Good (4) |  | Good (34) |
| Zhu, et al. (2020). (20) | RCT | Fair (3) | Fair (3) | Fair (3) | Poor (2) | Good (4) | Poor (2) | Fair (3) | Poor (2) | Good (4) |  | Fair (26) |
| Laccetti, et al. (2007). (21) | Secondary analysis of RCT | Fair (3) | Fair (3) | Good (4) | Good (4) | Fair (3) | Poor (2) | Poor (2) | Good (4) | Poor (2) |  | Fair (27) |
| Leal, et al. (2018). (22) | Qualitative analysis of EW texts | Good (4) | Poor (2) | Good (4) | Good (4) | Good (4) | Poor (2) | Good (4) | Good (4) | Fair (3) | Further research | Good (31) |
| Rose, et al. (2008). (23) | Secondary analysis of RCT | Poor (2) | Poor (2) | Fair (3) | Fair (3) | Good (4) | Poor (2) | Fair (3) | Fair (3) | Good (4) |  | Fair (26) |
| Rose, et al. (2009). (24) | Secondary analysis of RCT | Fair (3) | Poor (2) | Good (4) | Poor (2) | Good (4) | Very Poor (1) (2) | Good (4) | Good (4) | Good (4) |  | Good (28) |
| Radziewicz, et al. (2009). (25) | Secondary analysis of RCT | Fair (3) | Poor (2) | Fair (3) | Poor (2) | Fair (3) | Good (4) | Fair (3) | Fair (3) | Good (4) |  | Fair (27) |
| Garcia Perez and Dapueto (2014). (26) | Case study | Fair (3) | Poor (2) | Poor (2) | Good (4) | Poor (2) | Fair (3) | Fair (3) | Fair (3) | Fair (3) | Policy or practice implications | Fair (25) |
| Milbury, et al. 2018 (27) | ICE/Single arm experiment | Good (4) | Good (4) | Good (4) | Poor (2) | Fair (3) | Fair (3) | Fair (3) | Poor (2) | Good (4) |  | Good (29) |
| Pon, et al. (2010). (28) | Qualitative interview study | Fair (3) | Poor (2) | Poor (2) | Poor (2) | Poor (2) | Very Poor (1) (2) | Fair (3) | Poor (2) | Poor (2) | Policy or practice implications and further research | Fair (19) |
| Taylor, et al. (2016) (29) | Qualitative interview study | Good (4) | Fair (3) | Good (4) | Poor (2) | Good (4) | Fair (3) | Good (4) | Poor (2) | Good (4) |  | Good (30) |
| Tuck, et al. (2012). (30) | Quantitative - secondary analysis of RCT | Fair (3) | Fair (3) | Fair (3) | Poor (2) | Fair (3) | Poor (2) | Fair (3) | Poor (2) | Good (4) |  | Fair (25) |
| Milbury, K., et al. (2019). (31) | Not rated: protocol | | | | | | | | | | | |
| Milbury et al. (2018). (32) | Not rated: abstract | | | | | | | | | | | |

**Scoring:** Good = 4; Fair = 3; Poor = 2; Very Poor = 1

**Overall scoring key:** Good: ≥28 **;** Fair: 19-27 ; Poor: ≤18

**Quality appraisal instrument reference:**

Hawker S, Payne S, Kerr C, Hardey M, Powell J. Appraising the Evidence: Reviewing Disparate Data Systematically. Qual Health Res. 2002 Nov 1;12(9):1284–99.

**Scoring reference:**

en Boer K, de Veer AJE, Schoonmade LJ, Verhaegh KJ, van Meijel B, Francke AL. A systematic review of palliative care tools and interventions for people with severe mental illness. BMC Psychiatry [Internet]. 2019 Apr 3 [cited 2019 Dec 26];19. Available from: https://www.ncbi.nlm.nih.gov/pmc/articles/PMC6446277/

**References**

1. Arden-Close E, Gidron Y, Bayne L, Moss-Morris R. Written emotional disclosure for women with ovarian cancer and their partners: randomised controlled trial. Psychooncology. 2013 Oct;22(10):2262–9.

2. Averill AJ, Kasarskis EJ, Segerstrom SC. Expressive disclosure to improve well-being in patients with amyotrophic lateral sclerosis: a randomised, controlled trial. Psychol Health. 2013;28(6):701–13.

3. Bruera E, Willey J, Cohen M, Palmer JL. Expressive writing in patients receiving palliative care: a feasibility study. J Palliat Med. 2008 Feb;11(1):15–9.

4. de Moor C, Sterner J, Hall M, Warneke C, Gilani Z, Amato R, et al. A pilot study of the effects of expressive writing on psychological and behavioral adjustment in patients enrolled in a Phase II trial of vaccine therapy for metastatic renal cell carcinoma. Health Psychology. 2002;21(6):615–9.

5. Imrie S, Troop NA. A pilot study on the effects and feasibility of compassion-focused expressive writing in Day Hospice patients. Palliat Support Care. 2012 Jun;10(2):115–22.

6. Lloyd-Williams M, Cobb M, O’Connor C, Dunn L, Shiels C. A pilot randomised controlled trial to reduce suffering and emotional distress in patients with advanced cancer. J Affect Disord. 2013 May 15;148(1):141–5.

7. Lloyd-Williams M, Shiels C, Ellis J, Abba K, Gaynor E, Wilson K, et al. Pilot randomised controlled trial of focused narrative intervention for moderate to severe depression in palliative care patients: DISCERN trial. Palliative Medicine. 2018 Jan;32(1):206–15.

8. Low CA, Stanton AL, Bower JE, Gyllenhammer L. A randomized controlled trial of emotionally expressive writing for women with metastatic breast cancer. Health Psychol. 2010 Jul;29(4):460–6.

9. Manne S, Rubin S, Edelson M, Rosenblum N, Bergman C, Hernandez E, et al. Coping and communication-enhancing intervention versus supportive counseling for women diagnosed with gynecological cancers. - PsycNET. Journal of Consulting and Clinical Psychology. 2007;75(4):615–628.

10. Manne SL, Virtue SM, Ozga M, Kashy D, Heckman C, Kissane D, et al. A Comparison of Two Psychological Interventions for Newly-diagnosed Gynecological Cancer Patients. Gynecol Oncol. 2017 Feb;144(2):354–62.

11. Manne SL, Myers-Virtue S, Darabos K, Ozga M, Heckman C, Kissane D, et al. Emotional processing during psychotherapy among women newly diagnosed with a gynecological cancer. Palliat Support Care. 2017;15(4):405–16.

12. Virtue SM, Manne S, Criswell K, Kissane D, Heckman C, Rotter D. Levels of emotional awareness during psychotherapy among gynecologic cancer patients [Internet]. Palliative & supportive care. 2019 [cited 2020 Jul 20]. Available from: https://pubmed.ncbi.nlm.nih.gov/29880065/

13. Myers Virtue S, Manne SL, Darabos K, Heckman CJ, Ozga M, Kissane D, et al. Emotion episodes during psychotherapy sessions among women newly diagnosed with gynecological cancers: Emotion episodes during psychotherapy with cancer patients. Psycho-Oncology. 2015 Sep;24(9):1189–96.

14. Milbury K, Weathers Spw, Durrani S, Li Y, Whisenant M, J L, et al. Online Couple-Based Meditation Intervention for Patients With Primary or Metastatic Brain Tumors and Their Partners: Results of a Pilot Randomized Controlled Trial [Internet]. Journal of pain and symptom management. 2020 [cited 2020 Jul 20]. Available from: https://pubmed.ncbi.nlm.nih.gov/32061834/

15. Mosher CE, Duhamel KN, Lam J, Dickler M, Li Y, Massie MJ, et al. Randomised trial of expressive writing for distressed metastatic breast cancer patients. Psychol Health. 2012;27(1):88–100.

16. Porter LS, Keefe FJ, Baucom DH, Hurwitz H, Moser B, Patterson E, et al. Partner-Assisted Emotional Disclosure for Patients with GI Cancer: Results from a Randomized Controlled Trial. Cancer. 2009 Sep 15;115(18 Suppl):4326–38.

17. Steinhauser KE, Alexander SC, Byock IR, George LK, Olsen MK, Tulsky JA. Do preparation and life completion discussions improve functioning and quality of life in seriously ill patients? Pilot randomized control trial. J Palliat Med. 2008 Nov;11(9):1234–40.

18. Steinhauser KE, Alexander SC, Byock IR, George LK, Tulsky JA. Seriously ill patients’ discussions of preparation and life completion: an intervention to assist with transition at the end of life. Palliat Support Care. 2009 Dec;7(4):393–404.

19. Steinhauser KE, Alexander S, Olsen MK, Stechuchak KM, Zervakis J, Ammarell N, et al. Addressing Patient Emotional and Existential Needs During Serious Illness: Results of the Outlook Randomized Controlled Trial. J Pain Symptom Manage. 2017;54(6):898–908.

20. Zhu J, Hussain M, Joshi A, Truica CI, Nesterova D, Collins J, et al. Effect of creative writing on mood in patients with cancer. BMJ supportive & palliative care. 2020 Mar 1;10(1):64–7.

21. Laccetti M. Expressive writing in women with advanced breast cancer. Oncol Nurs Forum. 2007 Sep;34(5):1019–24.

22. Leal I, Milbury K, Engebretson J, Matin S, Jonasch E, Tannir N, et al. Interconnection: A qualitative analysis of adjusting to living with renal cell carcinoma. Palliat Support Care. 2018;16(2):146–54.

23. Rose JH, Radziewicz R, Bowmans KF, O’Toole EE. A coping and communication support intervention tailored to older patients diagnosed with late-stage cancer. Clin Interv Aging. 2008;3(1):77–95.

24. Rose JH, Bowman KF, Radziewicz RM, Lewis SA, O’Toole EE. Predictors of Engagement in a Coping and Communication Support Intervention for Older Patients with Advanced Cancer: Patient engagement in a coping and communication support intervention. Journal of the American Geriatrics Society. 2009 Nov;57:s296–9.

25. Radziewicz RM, Rose JH, Bowman KF, Berila RA, O’Toole EE, Given B. Establishing treatment fidelity in a coping and communication support telephone intervention for aging patients with advanced cancer and their family caregivers. Cancer Nurs. 2009 Jun;32(3):193–202.

26. García Pérez AI, Dapueto JJ. Case report of a computer-assisted psychotherapy of a patient with ALS. Int J Psychiatry Med. 2014;48(3):229–33.

27. Milbury K, Engle R, Tsao A, Liao Z, Owens A, Chaoul A, et al. Pilot Testing of a Brief Couple-Based Mind-Body Intervention for Patients With Metastatic Non-Small Cell Lung Cancer and Their Partners. Journal of Pain and Symptom Management. 2018 Mar 1;55(3):953–61.

28. Pon AKL. My Wonderful Life: A Board Game for Patients with Advanced Cancer. Illness, Crisis & Loss. 2010 Apr;18(2):147–61.

29. Taylor F, Combes G, Hare J. Improving clinical skills to support the emotional and psychological well-being of patients with end-stage renal disease: a qualitative evaluation of two interventions. Clin Kidney J. 2016 Jun;9(3):516–24.

30. Tuck I, Johnson SC, Kuznetsova MI, McCrocklin C, Baxter M, Bennington LK. Sacred healing stories told at the end of life. J Holist Nurs. 2012 Jun;30(2):69–80.

31. Milbury K, Li, Y, Durrani S, Liao Z, Yang C, Tsao A, et al. Results of a pilot randomized controlled trial: A couple-based meditation intervention for patients with metastatic lung cancer and their partners. | Journal of Clinical Oncology. Journal of Clinical Oncology. 2019;37(31_suppl):135–135.

32. Milbury K, Tsao AS, Liao Z, Owns A, Engle R, Gonzalez EA, et al. A research protocol for a pilot randomized controlled trial designed to examine the feasibility of a couple-based mind-body intervention for patients with metastatic lung cancer and their partners. Pilot and Feasibility Studies. 2018 Jan 24;4(1):37.
